# The clock gene *cycle* synchronizes circadian metabolic oscillations uniquely in reproductively active *Culex pipiens* females

**DOI:** 10.1038/s44324-026-00136-y

**Published:** 2026-10-06

**Authors:** Mizuki Yoshida, Matthew E. Wolkoff, Cheolho Sim, Megan E. Meuti, Matthias S. Klein

**Affiliations:** 1 Extremotolerance Biotechnology Group, Department of Biomaterial Sciences, Institute of Agrobiological Sciences, National Agriculture and Food Research Organization, 1-2 Owashi, Tsukuba, Ibaraki Japan; 2 Department of Entomology, The Ohio State University, 2021 Coffey Road, Columbus, OH USA; 3 Department of Veterinary Biosciences, The Ohio State University, 1900 Coffey Road, Columbus, OH USA; 4 Department of Biology, Baylor University, 101 Bagby Ave, Waco, TX USA; 5 Department of Animal Science, McGill University, 21,111 Lakeshore Road, Ste.-Anne-de-Bellevue, QC Canada

**Keywords:** Ecology, Ecology, Physiology, Zoology

## Abstract

The circadian clock regulates changes in daily metabolite abundance, coordinating animal activity, feeding and rest. The Northern house mosquito, *Culex pipiens*, also displays seasonal changes between periods of reproductive activity and overwintering diapause, regulated by changes in daylength. However, differences in metabolic oscillations between diapause and non-diapause have yet to be characterized. We hypothesized that reproductively active, non-diapausing mosquitoes would show large metabolic oscillations compared to diapausing mosquitoes. Our previous work demonstrated that CYCLE regulates insulin signaling, reproductive growth and olfaction, and that knocking out *cycle* causes a diapause-like state regardless of photoperiod, thus we predicted large-scale disruptions to the daily and seasonal metabolomes of *CpCyc*^*−/−*^ mosquitoes. We identified 27 metabolites showing highly synchronized oscillations in non-diapausing wildtype (WT) females, but only 11 oscillating metabolites in WT diapausing females. We also observed large-scale metabolic disruptions in *CpCyc*^*−/−*^ females reared under both long and short days, especially a loss of synchronicity between oscillations. This is the first report on differences in oscillating metabolites between diapausing and non-diapausing *Cx. pipiens*, and the impact of *cycle* on oscillating metabolites. This provides additional mechanistic insights as to how the circadian clock regulates daily and seasonal metabolic differences, with implications for insulin dysregulation and metabolic disorders.

## Introduction

Insects living in temperate regions enter prolonged states of arrested development, or diapause, to survive during harsh seasons such as winter^1,2^. Diapause involves large-scale metabolic changes, including fat accumulation prior to entering diapause and an overall suppression of metabolism that allows insects to conserve energetic resources for the duration of winter^3^. Diapause has been linked to the suppression of insulin signaling in many insects^4^, making it an excellent proxy for understanding metabolic changes associated with obesity, diabetes and lifespan extension in humans and other animals^5,6^.

Although some insects enter an obligate diapause at a specific life stage, most diapause-capable insects enter facultative diapause based on seasonal cues^2^. Photoperiod is a stable predictor for seasonal change, and many insects use the circadian clock to detect seasonal changes in daylength^7,8^. For example, knocking down the circadian clock gene *cycle* caused the bean bug, *Riptortus pedestris*, to enter a diapause-like state^9^, while knocking out *cycle* prevented the silkworm, *Bombyx mori*, from producing diapausing eggs^10^.

Previous studies have examined the metabolic differences between diapausing and non-diapausing flesh flies^11^, parasitic wasps^12^, Asian tiger mosquitoes^13^, cabbage white butterflies^14^, cotton bollworms^15^, wheat midges^16^, and Northern house mosquitoes^17,18^. Common features of diapause include the upregulation of cryoprotectants, including trehalose, glycerol and/or proline, and downregulation of most other amino acids^11,12,15,16^. Typical features of diapause, such as increased cold tolerance and suppressed metabolism, can be achieved by different mechanisms that are induced by global transcriptional reprogramming. Entry into diapause in *Cx. pipiens* involves differential expression of genes related to anaerobic metabolism, innate immunity, cold tolerance, and juvenile hormone synthesis^19,20^.

Differences in the oscillating circadian metabolome have been less thoroughly investigated, although a set of 18 shared metabolites displays rhythmic oscillations in multiple species (fruit flies, mice, and humans)^21^. Previously, Rhoades et al. found 34 metabolites that showed daily oscillations in *D. melanogaster*, seven of which continued to oscillate when transferred into constant darkness^22^. Surprisingly, per-null mutants of *D. melanogaster* that lacked a functioning clock still exhibited strong rhythmic differences in the abundance of 14 metabolites, but, unlike the wild-type flies, these oscillated with a 12-h period^22^. More recently, Malik et al. uncovered large-scale differences in the metabolome of wild-type flies and flies that had “short sleep” phenotypes caused by mutations in the genes *sleepless* and *fumin*^23^. These studies demonstrate that multiple metabolites oscillate on a daily basis in insects and that disruptions to the circadian clock can cause adverse metabolic consequences that may explain the shorter lifespans of *sleepless* mutants^24^ and higher metabolic rate in *fumin* mutants^25^. This mirrors work seen in humans where circadian disruptions caused through shiftwork, jet travel, sleep deprivation, and exposure to artificial light negatively impact the metabolome^26^.

Females of the Northern house mosquito, *Cx. pipiens*, transmit several deadly pathogens, including West Nile virus, St. Louis encephalitis virus, and canine heartworm^27^. Short daylengths cause females to enter an adult diapause where they cease host-seeking and blood-feeding^28,29^, characterized by suppression of insulin signaling and increased fat accumulation^30^ and lower levels of daily rhythmic activity^31^, with the exception of increased nighttime sugar feeding^32^. Our previous work demonstrated that diapausing *Cx. pipiens* had significantly higher levels of pimelic acid and choline and lower levels of L-alanine, histidine, and glycogen relative to non-diapausing and reproductively active females^17^. Additionally, we found that knocking down the clock genes *period*, *timeless*, and *cryptochrome2* prevented females reared in short-day, diapause-inducing conditions from entering diapause^33^, while knocking out the circadian clock gene *cycle* caused 70% of females that were reared in diapause-averting long days to enter a diapause-like state with small egg follicles^34^. Additionally, the CYCLE (CYC) protein has been identified as a master transcriptional regulator that binds directly to E-box elements in the promoter regions of genes governing reproductive growth, neuropeptide signaling, and olfaction^35^. Because CYC activity and abundance peak at dusk under long-day conditions, it likely serves as the primary driver for the transcription of downstream targets that initiate the transition between summer reproductive activity and winter dormancy.

Despite mounting evidence that the circadian clock in *Cx. pipiens* is involved in distinguishing long and short daylengths to coordinate the overwintering dormancy, we do not currently know which metabolites in *Cx. pipiens* oscillate under daily or seasonal conditions, and whether disruptions to the clock would similarly alter the metabolic profile of these mosquitoes. We hypothesized that non-diapausing and reproductively active mosquitoes would have a higher number of rhythmic and oscillating metabolites than diapausing females because non-diapausing females exhibit greater and more rhythmic locomotor and feeding activity^32^, a higher metabolic rate^18^, and Kang et al. previously found that transcripts associated with metabolism were downregulated in diapausing female *Cx. Pipiens*^19^. We also hypothesized that both long-day and short-day reared *CpCyc*^*−/−*^ females would exhibit large-scale disruptions to their circadian metabolome, similar to *D. melanogaster* with mutations in circadian clock genes^22^ and those associated with sleep cycles^23^ and because CYC regulates several important metabolic targets^35^. To test these hypotheses, we reared wild-type and *CpCyc*^*−/−*^ mosquitoes under both diapause-averting, long days and diapause-inducing short days. We then collected individuals at 4-h intervals over the course of 1 day and used NMR spectroscopy to identify and quantify the abundance of metabolites in female *Cx. pipiens*. Uncovering seasonal differences in the metabolome and the extent to which they are regulated by the circadian clock is important because the circadian regulation of metabolism is conserved across insects, humans and other animals^21^; thus, this study may further provide insights on how disruptions to the circadian clock (e.g., through shiftwork or exposure to light pollution) negatively impact metabolic health and contribute to disease development.

## Results

### Large-scale metabolic changes in diapausing and CpCyc^−/−^ mosquitoes

**Fig. 1:**
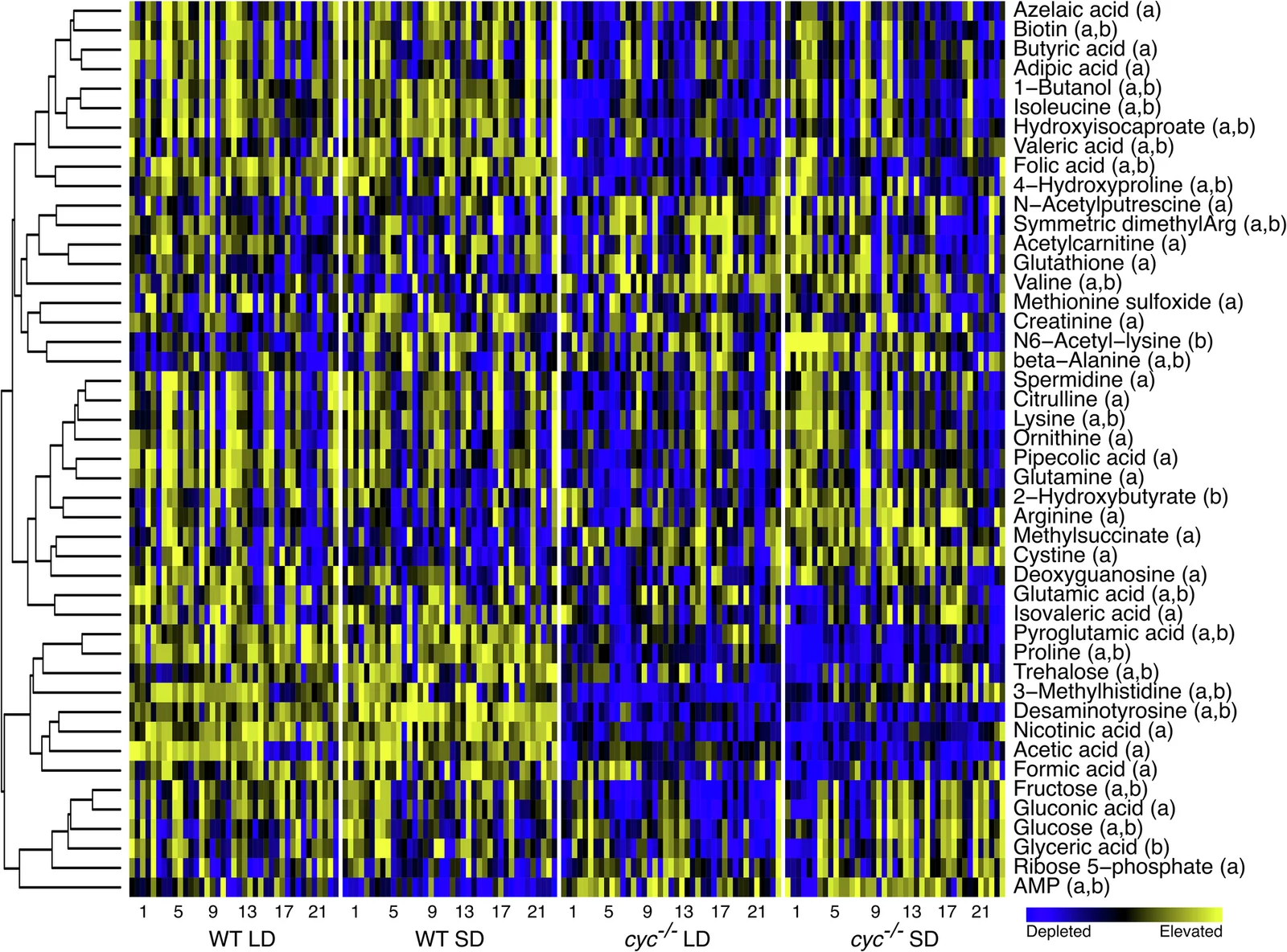
Heatmap of significant metabolites. Shown are metabolites differing between genotypes (**a**) and/or photoperiods (**b**). x-axis labels: LD long day, SD short day. Numbers (1–21) indicate time points in hours. Yellow/black/blue: high/intermediate/low concentration.

Principal component analysis (PCA) revealed that the overall metabolomic profiles of wild-type (WT) and *cycle*-null mutant (*CpCyc*^*−/−*^) mosquitoes under long- and short-day conditions showed considerable overlap, although partial separation of *CpCyc*^*−/−*^ samples was visible (Supplementary Fig. 1). Analysis of loadings (Supplementary Fig. 1B) revealed that this separation was driven by elevated levels of creatinine, methionine sulfoxide, and glucose in some *CpCyc*^*−/−*^ individuals. Further investigation using General linear models (GLM) revealed that 487 and 291 spectral bins significantly changed by genotype and photoperiod, respectively. Out of these signals, 44 and 30 metabolites were identified to be significantly correlated to genotype and photoperiod, respectively, including sugars, amino acids, and B vitamins (Fig. 1, Supplementary Table 1, and Supplementary Fig. 2).

Several metabolites, averaged over all time points, were significantly higher in short-day (diapause-inducing) WT as compared to long-day WT animals, including glucose, trehalose, and B vitamins biotin and folic acid. Conversely, we found that adenosine monophosphate (AMP) was depleted in diapausing females.

Compared to WT animals, many metabolites were on average significantly depleted in the *CpCyc*^*−/−*^ mutants, including organic acids such as acetic acid, formic acid, and butyric acid; B vitamins such as biotin, nicotinic acid, and folic acid; sugars such as trehalose and fructose; and amino acids such as proline and the branched-chain amino acid (BCAA) isoleucine. Glucose, creatinine, and amino acids cystine, arginine, and the BCAA valine were significantly upregulated in *CpCyc*^*−/−*^ mutants.

**Fig. 2:**
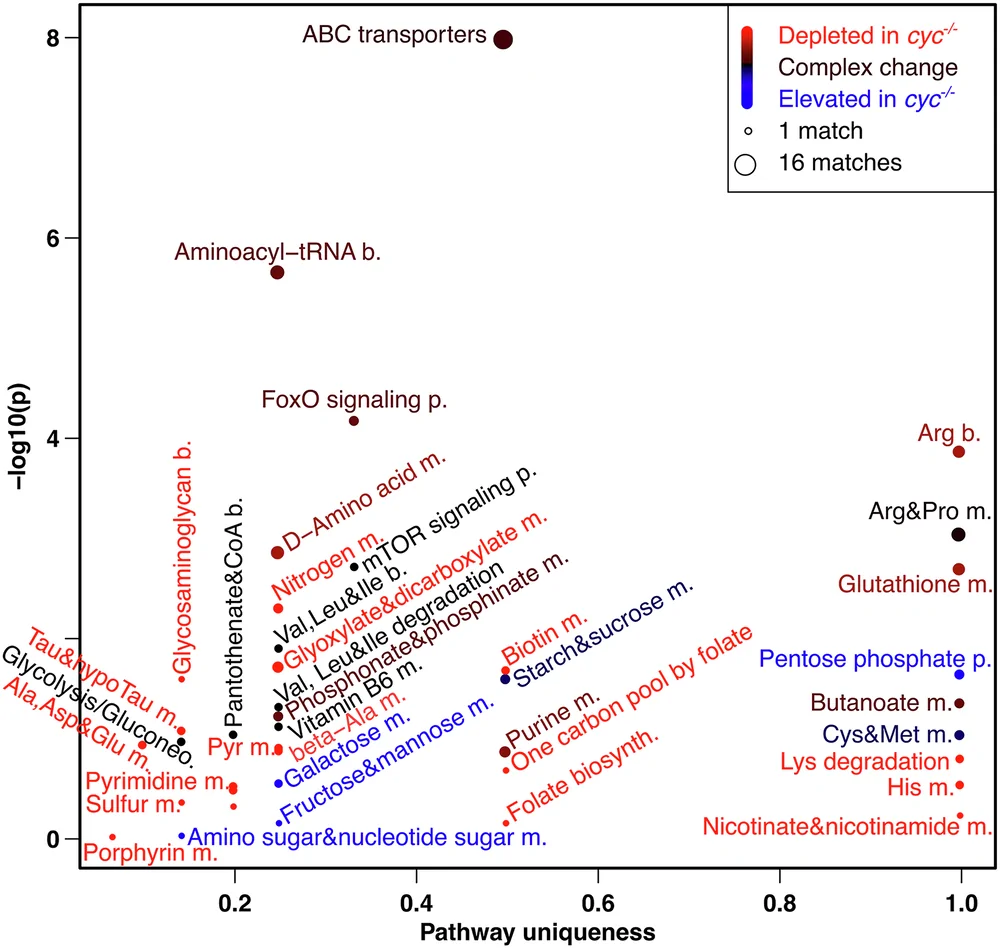
Pathway enrichment analysis for general differences between WT and *CpCyc*^*−/−*^ female mosquitoes. Colors represent average directions of change for significant metabolites within a pathway (red: mostly depleted, blue: mostly elevated, mauve/black: complex change with elevation of some metabolites and depletion of others). Note that non-significant metabolites in a pathway may follow a different pattern of depletion/elevation. m. metabolism, b. biosynthesis, p. pathway

Pathway enrichment analysis for the metabolites differentiating *CpCyc*^*−/−*^ from WT at the 0.05 level (FDR-corrected) revealed changes in ABC transporters, Aminoacyl-tRNA biosynthesis, FoxO signaling pathway, target of rapamycin (mTOR signaling) pathway, pentose phosphate pathway, glyoxylate and dicarboxylate metabolism, nitrogen metabolism, as well as biosynthesis and metabolism of various amino acids (Fig. 2). Unlike most pathways in the KEGG model, the ABC transporters pathway contains numerous specialized transporter proteins that share a function rather than sharing substrates or products. This leads to many potential hits among any list of significantly changing metabolites; thus, the apparent change in ABC transporters is likely spurious.

### Circadian metabolites in wild-type diapausing and non-diapausing females

**Fig. 3:**
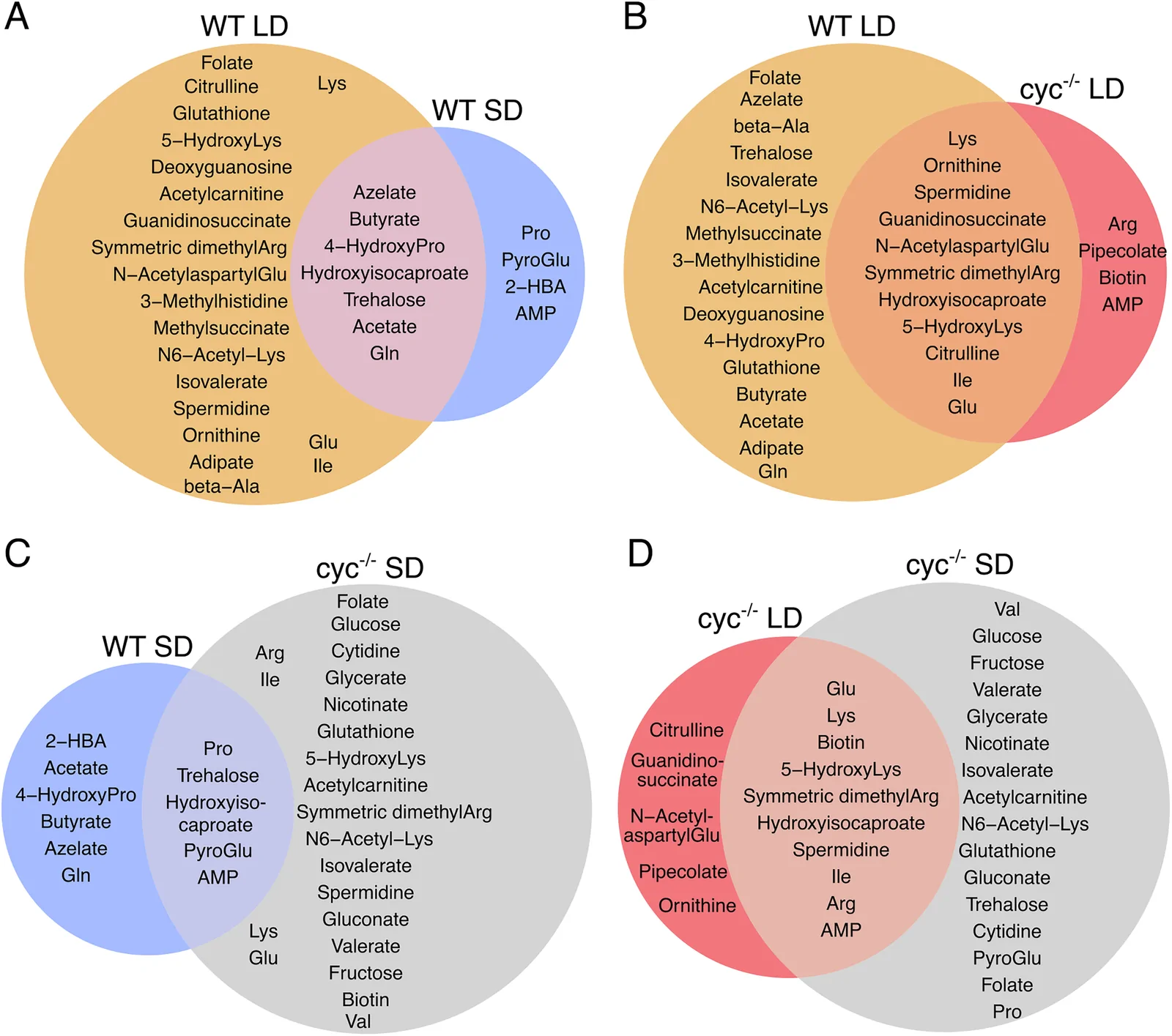
Metabolites that showed significant circadian oscillations. **A** WT females reared in long vs. short day conditions. **B** WT vs. *CpCyc*^*−/−*^ mutants reared in long-day conditions. **C** WT vs. *CpCyc*^*−/−*^ mutants reared in short-day conditions, **D** *CpCyc*^*−/−*^ mutant long day vs. short-day conditions.

Analysis of rhythmicity revealed that 129 bins showed significant circadian oscillations in WT mosquitoes during long-day conditions. These were identified as 27 unique metabolites (Supplementary Table 2 and Fig. 3A). When comparing the WT metabolite profiles from long-day conditions to short-day (diapause-inducing) conditions (Fig. 3A), far fewer metabolites (11) exhibit circadian rhythmicity in short-day conditions (*p* = 0.009), only seven of which are shared with long-day conditions. Of these seven, all but hydroxyisocaproic acid additionally showed significant differences in the oscillation peak times between long-day and short-day (Supplementary Fig. 3).

### Disrupted oscillation patterns in CpCyc^−/−^ mutants

Compared to WT mosquitoes, *CpCyc*^*−/−*^ female mosquitoes showed a completely different pattern of rhythmicity in long-day conditions—16 of the 27 metabolites that showed significant oscillations in WT did not show circadian activity in *CpCyc*^*−/−*^, and of the remaining 11 metabolites that showed rhythmicity in both genotypes, all but hydroxyisocaproic acid showed a significant change in rhythmic patterns (Fig. 3B and Supplementary Fig. 4). When comparing WT and *CpCyc*^*−/−*^ under short-day conditions (Fig. 3C and Supplementary Fig. 5), surprisingly, *CpCyc*^*−/−*^ females had far higher number of oscillating metabolites: 26 as compared to 11 in WT (*p* = 0.014), 5 of which oscillated in both WT and *CpCyc*^*−/−*^ females reared in short-day conditions, with AMP differing significantly in the observed pattern. Acetic acid oscillated in both long-day and short-day WT mosquitoes; however, no significant oscillations of acetic acid were observed in *CpCyc*^*−/−*^ mutants. Fig. 3D shows oscillating metabolites in *CpCyc*^*−/−*^ mutants under short-day and long-day conditions. It is noteworthy that largely different sets of metabolites exhibit oscillations under different photoperiods.

**Fig. 4:**
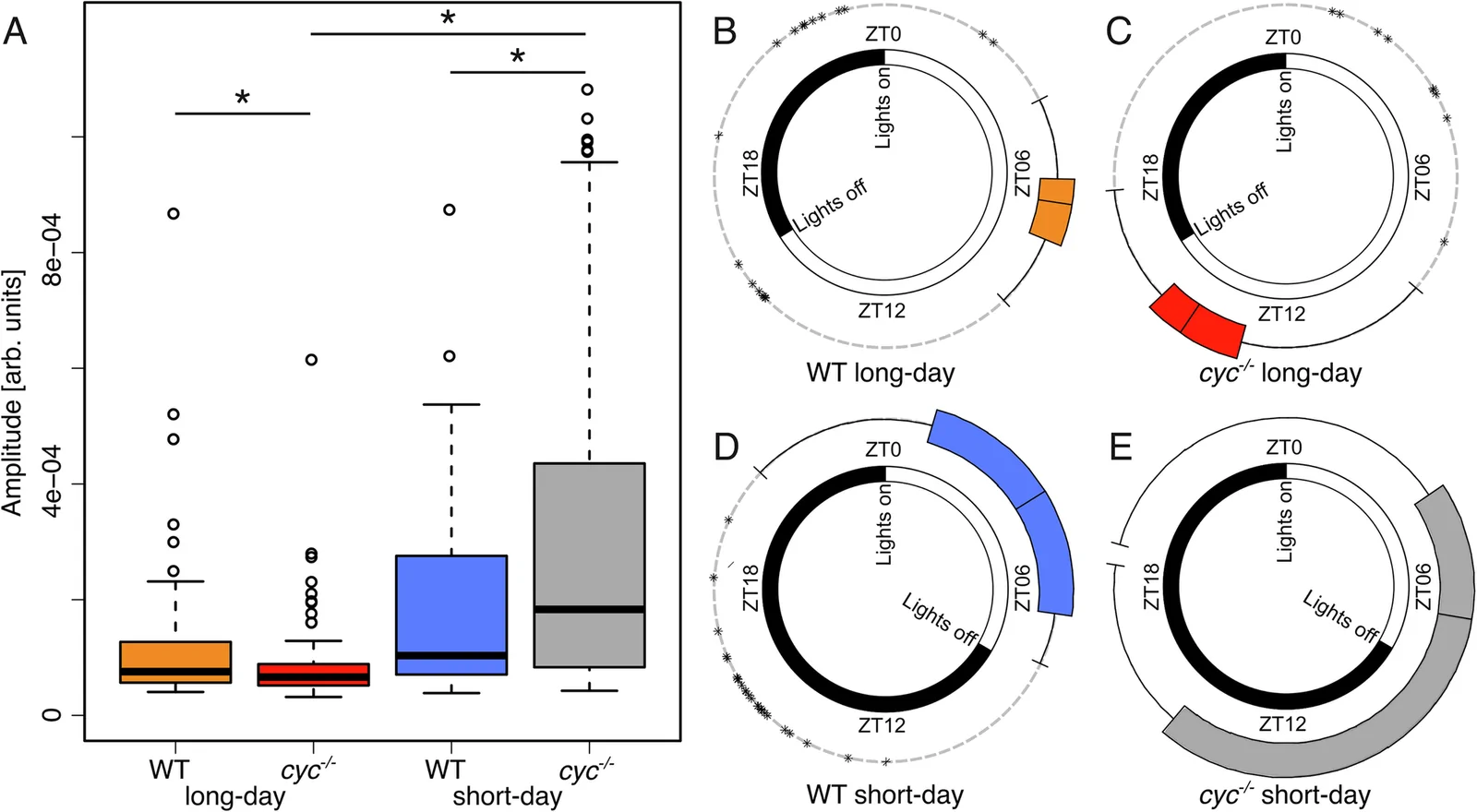
Comparison of the rhythmic parameters for all significantly oscillating metabolites. **A** Oscillation amplitude in arbitrary units, **B**–**E**: Oscillation peak times in ZT hours for WT long-day (**B**), *CpCyc*^*−/−*^ long day (**C**), WT short day (**D**), and *CpCyc*^*−/−*^ short day (**E**). Asterisks (*) indicate significant differences (FDR ≤20%). The upper limit of the amplitude axis was reduced to better visualize the main effects.

Figure 4A shows the amplitudes of significantly oscillating features for each group, reflecting the (absolute) change in metabolite concentrations during oscillations. Median amplitudes were generally higher in short-day conditions than in long-day conditions, on average 1.1-fold higher when combining WT and mutants. This difference was only significant for *CpCyc*^*−/−*^ mutants (corrected *p* = 1.3 × 10^−6^). The *CpCyc*^*−/−*^ mutation affected amplitudes differently during long-day and short-day conditions. In long-day conditions, *CpCyc*^*−/−*^ amplitudes were 12.3% lower than WT (corrected *p* = 0.032), while in short days, *CpCyc*^*−/−*^ increased amplitudes by 77.3% (corrected *p* = 0.032).

The distributions of peak times, the times of the daily maximum concentration values, are shown in Fig. 4B–E. Rayleigh tests of uniformity identified unimodal distributions of peak times for WT long-day and *CpCyc*^*−/−*^ long-day (corrected *p* = 1.8 × 10^−32^ and 3.3 × 10^−21^), indicating that in these conditions, most oscillating metabolites exhibited similar patterns. For WT short-day, the p-value for uniformity was 1.2 × 10^−3^ and did not reach significance for *CpCyc*^*−/−*^ short-day, indicating that in these cases, oscillations were not very synchronized and peak times were spread out across the day. The abundance of oscillating metabolites shows a narrow and consistent peak centered around mid-day (median ZT 6.59 h) in WT females reared in long-day conditions (Fig. 4B). However, in *CpCyc*^*−/−*^ females reared in long-day conditions (Fig. 4C), the peak time of oscillating metabolites shows that the median peak time in the mutants was not around mid-day as in WT, but close to lights-off time (median ZT 14.3 h). In addition, the peak times showed a much broader distribution, indicating a breakdown of coordinated rhythmicity; this difference was significant (*p* < 0.001). In short-day conditions, the peak times of oscillating metabolites in both WT (Fig. 4D) and *CpCyc*^*−/−*^ (Fig. 4E) female mosquitoes show a broad distribution, with an interquartile range between 4 and 16 h, indicating that metabolites oscillated with greater amplitudes and variances as compared to long days. Interestingly, under long-day conditions, the WT mosquitoes had a median peak time around mid-day (ZT 3.87 h), while mutants, again, had a median peak time close to lights-off time (ZT 6.62 h, *p* < 0.001). The peak times of short-day and long-day WT mosquitoes were significantly different (*p* < 0.001). In *CpCyc*^*−/−*^ mosquitoes, this difference was significant, too (*p* < 0.001). Closer examination of these results revealed that in *CpCyc*^*−/−*^ under short-day conditions, sugar molecules such as glucose and fructose exhibit oscillating behavior. In contrast, sugar molecules did not show circadian patterns in the other groups. A summary of oscillating metabolites and suggested biological roles can be found in Supplementary Table 6.

**Fig. 5:**
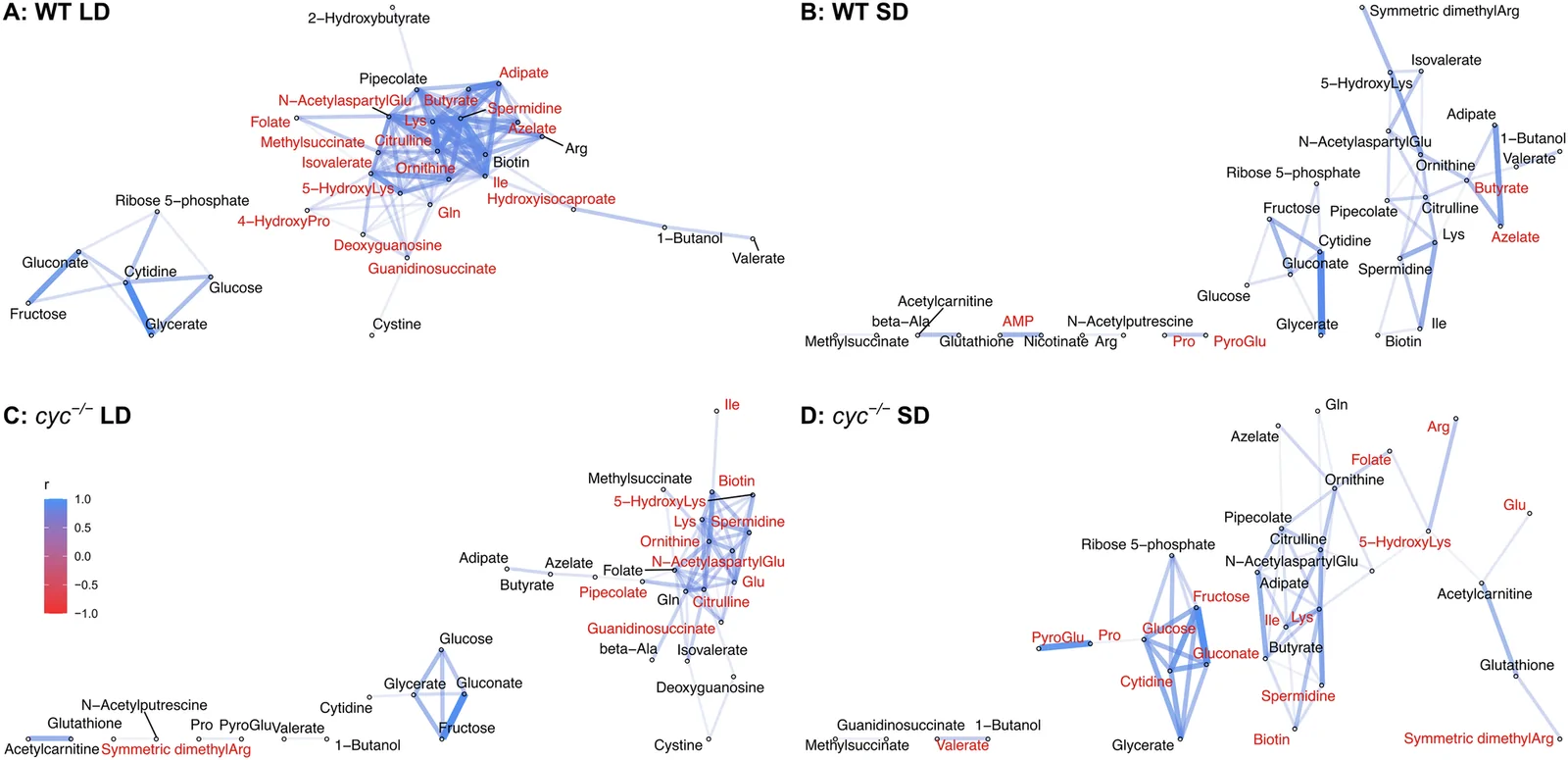
Correlation network analysis. Molecules that are significantly correlated are connected; the line color and width indicate the strength of correlation (blue = positive correlation; red = negative correlation). Metabolites that were not significantly correlated were omitted from the plot. Names of significantly oscillating metabolites are given in red font. **A** WT long day, **B** WT short day, **C** *CpCyc*^*−/−*^ long day, **D** *CpCyc*^*−/−*^ short day.

To further investigate the apparently highly synchronized behavior of metabolites, especially in WT females in long-day conditions, we calculated correlation networks for each combination of conditions (Fig. 5). In WT long-day conditions, 131 pairs of metabolites showed significant correlations, this dropped to only 44 in WT short-day. *CpCyc*^*−/−*^ long-day showed only about half of the correlations compared to WT (68), and 58 in *CpCyc*^*−/−*^ short-day. For WT long-day, a large cluster of highly connected metabolites is visible to the top right in Fig. 5A, containing most of the significantly oscillating metabolites of this group. These metabolites, including amino acids such as arginine, isoleucine, and lysine, and B vitamins such as folic acid and biotin, thus exhibit highly synchronized circadian oscillations in WT females reared in long-day conditions. A separate cluster of positively correlated molecules that did not oscillate is visible to the lower left; these include sugars such as glucose and fructose, related metabolites such as gluconic acid and glycerate, as well as RNA-related metabolites such as cytidine and ribose-5-phosphate. The correlations between these non-oscillating metabolites seem to stem from basic metabolic regulatory pathways and do not seem to be regulated in a circadian pattern in WT long-day conditions.

When comparing the 2-cluster network observed in WT long-day females to the network observed in WT females reared in short-day conditions (Fig. 5B), the non-circadian sugar/RNA cluster is largely identical to that in the WT long-day conditions, demonstrating that relationships between sugar and RNA molecules is a conserved cluster of metabolic correlations. However, the large cluster of oscillating amino acids visible in long-day conditions is decimated and broken into four smaller clusters, indicating that molecules such as methylsuccinic acid, arginine, glutamine, etc., are no longer synchronized. Only two of the remaining metabolites in the large cluster were found to oscillate in WT short-day, butyric acid and azelaic acid, although their rhythmic behavior seems to be inversed as compared to long-day (Supplementary Fig. 3). Interestingly, none of the molecules that exhibited circadian oscillations uniquely in WT short-day conditions (AMP, Pro, pyroglutamic acid) were part of the large cluster of correlated metabolites (top right of Fig. 5B), but three out of four of them formed small, unconnected clusters (lower left of Fig. 5B). This indicates that although AMP, Pro, pyroglutamic acid oscillated, they were not synchronized with each other. A similar picture is observed in *CpCyc*^*−/−*^ that were reared in long-day and short-day conditions (Fig. 5C, D), highlighting that mutating *cyc* disrupted synchronous oscillations in the metabolome that were characteristic of WT long-day conditions.

## Discussion

In this study, we carefully measured water-soluble metabolites extracted from the headless bodies of wild-type (WT) and *CpCyc*^*−/−*^ females of *Cx. pipiens* that were reared both in long-day, diapause-averting conditions, and in short-day, diapause-inducing conditions. Overall, we found that multiple metabolites oscillate in a highly synchronized manner in WT long-day, non-diapausing females, and that fewer metabolites showed rhythmic oscillations in WT short-day diapausing females. Additionally, we found that the mutation in the clock gene *cycle* disrupted rhythmic oscillations of metabolites, especially in long-day, diapause-averting conditions. This is consistent with our hypotheses as well as our previous results, where we found that most *CpCyc*^*−/−*^ females were in diapause regardless of whether they were reared in long-day or short-day conditions^34^.

This is the first study to report large-scale differences in oscillating metabolites between diapausing and non-diapausing insects. Although this is the first investigation of differences in the daily metabolome of diapausing and non-diapausing insects, our metabolic data are consistent with overall lower levels of locomotor activity^31^, downregulation of metabolism-associated pathways^19^ and lower levels of glycogen oxidation^18^ in diapausing females of *Cx. pipiens* and metabolic suppression that characterize diapause^3^.

A detailed assessment of rhythmic behavior of metabolites by genotype and photoperiod revealed that a large number of metabolites showed highly synchronized circadian oscillations in WT mosquitoes under long-day conditions, with a median peak time around mid-day (Fig. 4B). Several of the metabolites that we identified as oscillating in WT females in long-day conditions also oscillate in *D. melanogaster*, mice, and humans^21^, namely acetylcarnitine, lysine, and isoleucine. Thus, our data and previous studies demonstrate that there is a high degree of conservation among the daily metabolome of animals.

In contrast to non-diapausing females, a much smaller number of metabolites oscillated in WT females that were reared in short-day, winter-like conditions. In addition, these oscillations were largely not synchronized in diapausing females, as evidenced by large fluctuations in oscillation peak times (Fig. 4D) and a lack of correlation between the metabolites (Fig. 5B). Still, similar to the long-day reared WT mosquitoes, median peak times in short-day WT mosquitoes were around mid-day. Please note that mid-day corresponds to ZT04 in short days but ZT08 in long days. This robust shift in oscillation patterns is likely to be driven by a combination of overarching physiological adaptations and the photoperiod-dependent roles of the core clock machinery. One such physiological adaptation is the switch to feeding on sugar primarily at the transition from lights-on to lights-off in non-diapausing females, while diapausing females are feeding on sugar at low levels all night long^32^. These differences in feeding behavior are likely to contribute to the coordinated and narrow peak in metabolites in the non-diapausing females (several hours after nutrient ingestion) and a broader peak in diapausing females. Given the long time span of 14 h from lights-off to the observed median peak time, circadian regulation of metabolite levels is likely to contribute to this observed pattern as well. Our results are thus consistent with previous studies demonstrating that diapausing females of *Cx. pipiens* exhibit higher levels of nighttime sugar feeding^32^, reduced circadian locomotor activity^31^, greater synthesis of lipids and lower levels of glycogen metabolism^18^, and overall changes in gene expression consistent with a metabolic switch from host-seeking, blood-feeding and reproduction to nutrient storage and increased stress tolerance^19,20,36^.

We also observed that WT mosquitoes in long-day conditions exhibit higher numbers of oscillating metabolites than in short-day conditions, suggesting that CYC’s regulatory function is fundamentally photoperiod-dependent. In long-day environments, CYC likely operates as a transcriptional activator that promotes synchronous metabolic rhythms, whereas in short-day conditions, it appears to adopt a repressive role whose loss in mutants consequently disinhibits and increases oscillatory outputs. This contextual shift aligns with the dynamics of circadian neuronal networks, where CYC may differentially influence evening (E) oscillators—that dominate and drive rhythms in long-day conditions—and morning (M) oscillators, which dominate in rhythms in short-day conditions and where CYC may act to constrain rhythmicity. Ultimately, the overall extent of metabolic oscillation is shaped by a complex interplay between seasonal changes in daylength, the photoperiod-specific roles of core clock components like CYC, and the shifting balance within underlying neuronal oscillator networks.

Unlike the WT females that showed synchronized oscillations in long-day but not in short-day conditions, *CpCyc*^*−/−*^ mutants showed non-synchronized oscillations both in long-day and short-day conditions, with little resemblance to the profiles of the WT oscillating metabolites. Median peak times in the *CpCyc*^*−/−*^ mutants corresponded to lights-off time both under long-day and short-day conditions, indicating regulation by direct changes in light conditions rather than regulation through the circadian clock.

Acetic acid oscillated in both long-day and short-day WT mosquitoes; however, no significant oscillations were observed in *CpCyc*^*−/−*^ mutants. Together with the significant depletion in acetic acid in mutants described above, the disrupted oscillation of acetic acid observed in *CpCyc*^*−/−*^ mutants is particularly noteworthy when integrated with recent genetic data. CYC has been identified as a direct transcriptional regulator of ionotropic receptor 75a-like (Ir75a), a sensory receptor specifically tuned to volatile acidic compounds, including acetic acid^35^. The concurrent loss of the internal metabolite and the disruption of its primary sensory receptor in mutants suggest that the circadian clock facilitates a highly synchronized network, coordinating internal metabolic abundance with external sensory perception to regulate time-of-day specific host-seeking and blood-feeding behaviors in *Cx. pipiens*.

A cluster of correlated sugar metabolites did not oscillate in WT mosquitoes under long or short days, indicating proper glycemic control through insulin secretion. In *CpCyc*^*−/−*^ mutants under short-day conditions, however, significant rhythmicity is observed for this cluster. This may be attributable to an increased rate of late-night sugar feeding in the mutants^32^ combined with disturbed insulin signaling through the loss of CYCLE in the mutants.

PCA analysis identified elevated levels of glucose, creatinine, and methionine sulfoxide (MetSO) in *CpCyc*^*−/−*^ mutants. The generally elevated glucose levels in mutants provide physiological evidence for a disrupted metabolic switch governed by the photoperiodic clock. In wild-type mosquitoes, CYC drives the production of insulin-like peptide 1 (ILP-1); under long-day conditions, high ILP-1 levels deactivate the FoxO transcription factor, thereby facilitating reproductive development^35^. In *CpCyc*^*−/−*^ mutants, the loss of CYC-mediated regulation likely suppresses insulin signaling. This suppression allows FoxO to remain constitutively active, driving the metabolic switch toward nutrient storage, fat accumulation, and elevated glucose levels that are characteristic of diapause. Thus, elevated glucose in the mutants may indicate dysregulation of insulin signaling pathways.

Elevated creatinine is usually connected to either higher muscle mass or elevated physical activity. A previous study in the plant bug, *Iphita limbata*, found elevated levels of hemolymph creatinine and total creatinine during diapause, while creatinine in the fat body was depleted during that period^37^. This aligns with our finding that creatinine is also elevated in diapausing WT female *Cx. pipiens*, although this trend did not reach significance. Although *CpCyc*^*−/−*^ mosquitoes exhibit similar activity levels under light:dark or diel conditions, mean activity levels in *CpCyc*^*−/−*^ mosquitoes are higher when they are exposed to constant darkness (D:D)^34^. In addition, our previous study revealed that while the mean activity levels of both WT and *CpCyc*^*−/−*^ mosquitoes were similar when they were exposed to diel conditions, WT mosquitoes were primarily active in punctuated bursts at transitions between photophase and scotophase and rested during photophase, whereas the mutants had lower peak locomotor levels but were active over longer periods of time with no or limited rest periods. These differences in the overall activity of *CpCyc*^*−/−*^ mutants may have contributed to persistently elevated creatinine levels.

MetSO, an oxidatively damaged form of the proteinogenic amino acid methionine (Met), was generally elevated in *CpCyc*^*−/−*^ mutants. In *Drosophila*, the enzymatic reduction of MetSO to Met is dependent on activation of the FoxO pathway^38^. The observed elevated levels of MetSO indicate lower levels of reduction to Met in *CpCyc*^*−/−*^ mutants, which could be linked to the changes in FoxO identified in the pathway enrichment analysis. MetSO itself has also been described to play a vital role during diapause regulation in the silkworm *Bombyx mori*, such that females that were exposed to short photoperiods and produced diapausing eggs had higher levels of MetSO in their hemolymph than long-day-exposed females^39^.

A more granular analysis using linear models revealed a large number of metabolic changes. A large number of metabolites exhibited significant change in average abundance between short-day and long-day conditions. As a notable example, the average abundance of AMP across all time points was depleted in diapausing females, while Sapre et al. found that *D. melanogaster* exposed to short days and lower temperature (20 °C) had a higher abundance of AMP in their brains^40^. Of note, our study measured the abundance of metabolites in headless mosquitoes, so these results cannot be directly compared.

Analyses revealed an even larger number of metabolites that showed significant changes in average abundance in the *CpCyc*^*−/−*^ mutants compared to WT. For example, we observed a reduction of average proline in *CpCyc*^*−/−*^ mutants. Proline is the most abundant amino acid in *Cx. Pipiens*^41^ and can serve as an alternative energy source, for example, during the flight of female yellow fever mosquitoes, *A. egypti*^42^. Higher levels of proline have been found in overwintering *Cx. pipiens*, but not at levels that would create a truly cryoprotectant effect as in other insects^43^. The observed reduction of proline we observed in *CpCyc*^*−/−*^ may thus in part be explained by a higher sustained level of physical activity in mutants, depleting the proline levels as an energy source for flight, as discussed for creatinine above. However, proline is largely derived from body fat rather than dietary sources in tsetse flies^44^, hence, reduced levels of proline may indicate depleted lipid storage and/or altered lipid metabolism in *CpCyc*^*−/−*^ mutants. While this may seem likely given the changes in insulin signaling mentioned earlier, further studies are necessary to characterize changes in lipid storage and composition in *CpCyc*^*−/−*^ mutants. Malik et al. similarly observed disruptions in proline levels in short sleep mutants of *D. melanogaster*, indicating a general connection of proline to circadian regulation^23^.

In addition, the average abundance of B vitamin-related metabolites folic acid, nicotinic acid, and biotin were all significantly lower in *CpCyc*^*−/−*^. Disruptions to nicotinate and nicotinamide signaling have also been reported in short sleep mutants of *D. melanogaster*^23^, highlighting the relevance of B vitamins in circadian regulation. These molecules have all been shown to be essential for mosquito survival, stress tolerance, and longevity^45^, so the observed downregulation in *CpCyc*^*−/−*^ mutants is likely to negatively impact stress tolerance.

Metabolic pathway analysis pointed to major changes in the FoxO and mTOR signaling pathways in *CpCyc*^*−/−*^ mutants. FoxO, the forkhead box O transcription factor pathway, has been previously identified as a crucial regulator for diapause^46^. mTOR is crucial for modulating entrainment, synchronization and autonomous oscillation of circadian clocks, further supporting a link between circadian and diapause regulation^47^. Therefore, the disruption of FoxO and mTOR pathways in *CpCyc*^*−/−*^ is consistent with the reduced capacity of the mutants to measure seasonal and daily time. FoxO is also related to insulin signaling pathways, further providing context for the observed changes in glucose and MetSO levels in *CpCyc*^*−/−*^. These pathway disruptions are logically supported by the recent discovery that CYC directly regulates critical reproductive accelerators, including allatotropin and Krüppel homolog 1 (Kr-h1)^35^. Allatotropin stimulates the synthesis of Juvenile Hormone III (JH III), which is essential for reproductive development, while Kr-h1 regulates photoperiodic reproductive plasticity. In the absence of CYC, the transcriptional drive for these pathways is likely lost. This provides a mechanistic explanation for why *CpCyc*^*−/−*^ mutants fail to maintain a non-diapausing, reproductive metabolic profile under long days, and instead default to reproductive dormancy. Therefore, disruption in both pathways is consistent with our previous findings that *CpCyc*^*−/−*^ mutants are unable to correctly interpret long or short-day photoperiods such that the majority of mutant females enter a diapause-like state regardless of photoperiod^34^.

While our results are significant and interesting, we acknowledge that NMR spectroscopy has a relatively low sensitivity, especially when working with limited sample amounts, such as single mosquitoes. One option for improving sensitivity would be to pool extracts of multiple insects to increase metabolite concentrations. We decided against this approach to allow analysis of individual metabolic differences and instead carefully optimized sample preparation protocols, NMR parameters, and data analysis workflow, and used a high-end spectrometer, to limit the effects of the intrinsic sensitivity issues. The fact that we were able to identify numerous metabolites in the extracts that mirror previous research on diapause and/or circadian metabolomes makes us confident in the appropriateness of our approach. A related consideration is our use of extracts from the headless bodies of *Cx. pipiens*. While this preserved individual variance for our current methodology, recent transcription profiling emphasizes that diapause-related metabolic pathways are highly localized^48^. When using homogenates, metabolites from different tissues, body compartments, and the hemolymph are mixed, potentially averaging out and masking tissue-specific rhythmic patterns. On the other hand, this might also distort parameters of rhythmic patterns and create rhythmic artifacts. Consequently, future metabolomic analyses should isolate specific tissues, such as the fat body, head, or hemolymph, to precisely map where these synchronized circadian oscillations occur and how they break down in *CpCyc*^*−/−*^ mutants.

Although several researchers have characterized large-scale changes in transcription and metabolism in mosquitoes after ingesting a bloodmeal^49–52^, these are usually compared to a single timepoint in sugar-fed mosquitoes that are not necessarily time- or age-matched. As our results demonstrate that the metabolome of sugar-fed, non-diapausing females exhibits substantial changes throughout a 24 h period, future studies that measure differences in gene expression and metabolism in mosquitoes and other blood-feeding arthropods in response to a bloodmeal could potentially benefit from including time-matched, sugar-fed individuals, as this will enable researchers to distinguish differences unique to the digestion of vertebrate blood and vitellogenesis from naturally occurring daily differences in transcript abundance and metabolism. Additionally, studies that compare diapausing and non-diapausing insects should use similar time-matched controls to account for daily differences in transcription, metabolism, and behavior.

The convergence of our circadian metabolomic profiles with previously identified CYCLE (CYC) transcriptional targets reveals a highly synchronized, multi-tiered regulation of mosquito behavior, stress tolerance, and reproductive energy allocation. Our pathway enrichment analysis highlights profound disruptions in excitatory amino acid metabolism (including arginine, proline, and glutamate) within *CpCyc*^*−/−*^ mutants, directly paralleling the loss of CYC-mediated transcription for key synaptic and neurotransmitter receptors (nAChRa, Oct/Tyr R, Neuroligin-1) and providing a mechanistic basis for circadian behavioral deficits. Beyond locomotion and learning, the combined multi-omics data emphasizes the clock’s critical role in priming redox balance and olfactory feedback; disruptions in nicotinate and glutathione metabolism physically manifest the compromised stress defense expected from the loss of CYC-regulated immune factors (*takeout*, *Eip74EF*), while the specific depletion of volatile acidic metabolites (butyric and acetic acids) perfectly mirrors the transcriptional loss of their primary sensory receptor, Ir75a. Ultimately, the uncoupling of energy metabolites within the TCA cycle and starch and sucrose pathways reflects a systemic halt of reproductive anabolism, demonstrating that the loss of CYC-driven insulin signaling allows the FoxO transcription factor to remain constitutively active, thereby overriding reproductive accelerators like Kr-h1 and forcing the metabolic state to default toward nutrient storage and diapause.

In conclusion, our study demonstrates that there are largescale differences in the metabolome between reproductively active, non-diapausing mosquitoes and stress-tolerant diapausing mosquitoes, and that mutations to the circadian clock disrupted both daily and seasonal changes in metabolite levels. Specifically, we observed a high number of metabolites oscillate in a highly coordinated and synchronous manner in WT non-diapausing female mosquitoes, and that most of these metabolites no longer oscillated in diapausing females. Although we are the first to compare daily differences in the metabolome of diapausing and non-diapausing insects, several of the metabolic differences that we uncovered between diapausing and non-diapausing *Cx. pipiens* are consistent with findings in other insects^11–14,16,40^. Additionally, consistent with previous research with *per*-null and sleep mutants in *D. melanogaster*^22,23^, we found that disrupting the circadian clock through mutations in a core clock gene, *cycle*, also altered the metabolic profile in similar ways with disruptions to nicotinic acid, glutamate, arginine and proline. Our study provides important insights into how the metabolome of animals can respond to both daily and seasonal changes in light:dark cues, and further demonstrates that the circadian clock is involved in regulating both daily and seasonal changes in metabolism in insects and other animals. This is significant because several core metabolites that oscillate in non-diapausing WT *Cx. pipiens* also oscillate in fruit flies, mice and humans^21^, highlighting the connections between the current study, human circadian metabolism, and metabolic health.

## Methods

### Insects

*Culex pipiens* from the OSU Buckeye line were used as wildtype (WT). The *cycle*^−/−^ mosquito line of *Cx. pipiens* (hereafter *CpCyc*^*−/*−^) was generated from the same lab strain in a previous study^34^. Mosquitoes were reared at 18 °C under long-day, summer-like conditions (light:dark = 16:8 h) or under short-day, winter-like conditions (light:dark = 8:16 h). Data loggers in the incubators confirmed that there were no substantial temperature differences between the photophase and scotophase within either incubator (Long-day incubator; photophase: 18.3 ± 0.04 °C [average±stdev]; scotophase: 18.3 ± 0.05  °C; Short-day incubator; photophase: 18.1 ± 0.3 °C; scotophase 18.3 ± 4 °C). For the remainder of this manuscript, time is given in Zeitgeber [ZT] time, with 0 hours (ZT0) corresponding to the time when lights turned on, and ZT16 and ZT08 representing when lights turned off for the long and short photoperiods, respectively. Larvae were reared in plastic containers filled with reverse osmosis (RO) water. All larvae were fed on ground fish flakes (Tetramin tropical fish flakes, Tetramin, Tetra, Mulle, Germany)^53^. Adults were reared in cages (30 cm^3^) and provided RO water and 10% sucrose solution. To minimize variability, mosquitoes for each photoperiod were reared in the same incubators, and WT and *CpCyc*^*−/−*^ mosquito cohorts were reared simultaneously.

### Extracting metabolites

One-week-old, WT and *CpCyc*^*−/−*^ female mosquitoes that had been reared in long-day and short-day conditions were collected at 4-h intervals starting one hour after the lights had turned on (ZT01) and for five additional time points (ZT05, ZT09, ZT13, ZT17, and ZT21). For the present study, eight individuals were euthanized for each genotype, rearing condition, and timepoint by placing female mosquitoes in a −70 °C freezer. The heads of all mosquitoes were removed for separate analysis, and the headless mosquitoes were placed in a 2-mL tube with 750 mg of ceramic beads and homogenized in 400 µL cold methanol and 85 µL water. The homogenate was transferred to a new tube with 400 µL chloroform and 200 µL water, vortexed and then centrifuged at 2000 rcf for 5 min at 4 °C. The aqueous layer was removed, evaporated, resuspended in deuterium oxide (heavy water) containing trimethylsilylpropanoic acid (TSP) and boric acid, and pH-adjusted to 7.4.

### Metabolomic analysis

Metabolites in mosquito samples were measured using Nuclear Magnetic Resonance (NMR) spectroscopy. 1D ^1^H NOESY spectra were obtained for all individual aqueous extracts, and a ^1^H-^13^C HSQC spectrum was acquired for a pooled sample on an Ascend 850 MHz spectrometer equipped with a cryogenic probe (Bruker BioSpin, Billerica, MA, US). Topspin 4.5.0 (Bruker) was used for automated phase and baseline correction. Several spectra exhibited broad, lipid-like signals, potentially caused by incomplete separation of the aqueous and lipophilic phases; these spectra were excluded from further analysis. The untargeted approach of spectral binning was chosen, where each spectrum is divided into equally sized sections before analysis. NMR spectra were binned using the R package mrbin 1.9.4^54^, using a bin width of 0.003 ppm in the area between 9.5 and 0.5 ppm. Broader bins were manually defined for signals that visibly shifted between spectra. Besides the residual water signal, signals of methanol and ethanol were excluded, as these were introduced during extraction. We also excluded all sucrose signals as we suspected that most of the sucrose represented undigested sugar in the mosquito gut. Noise signals were automatically removed, and data were scaled using probabilistic quotient normalization (PQN) to correct for differences in sample mass and extraction efficacies. This resulted in a dataset of 813 features (spectral bins) for 161 samples. It should be noted that most metabolites occupy more than one bin, while a single bin may contain signals from multiple (overlapping) metabolites. Signals of interest were identified using public databases (HMDB and BMRB) and the HSQC spectrum.

### Data analysis

All data were analyzed with R version 4.5.2 (R Core Team, 2025). Principal Component Analysis (PCA) models were generated to visualize metabolic data and screen for outliers regarding data quality. To analyze metabolic differences between daylengths and genotypes, general linear models (GLM) were generated with these two factors for each bin. For GLM analysis only, data from each individual feature (bin) was unit variance scaled for improved interpretation. Resulting *p* values were corrected using FDR control at the 20% level. Pathway enrichment analysis was performed as previously described^17^. Briefly, a pathway map of *Cx.* *pipiens* *pallens* was downloaded (KEGG code cpii)^55^, and the number of matches per pathway was assessed by Fisher’s exact test corrected by FDR. A pathway uniqueness score was calculated to rate how unique specific metabolites were for a pathway^17^.

For analysis of circadian rhythmicity, the R package circacompare 0.2.0 was used^56^. This method models a cosinusoidal curve to experimental data using a parametrized approach, so differences between experimental conditions can be tested for the parameters mesor (the “baseline” level), amplitude (the amount of periodic change), and phase (the timepoint of the maximum variable level). For each bin, separate models were fitted for each combination of daylength and genotype using the circa_single function. The presence of rhythmicity was tested, and p-values were corrected for FDR at the 20% level. Phase uniformity of these significantly oscillating signals was tested using the Rayleigh test of uniformity from the circular package 0.5–2 and corrected using FDR. The number of significantly oscillating metabolites were compared using Pearson’s chi-squared test. Peak times and amplitudes of signals that oscillated were compared in a planned contrast analysis for four pairwise comparisons: WT long-day-WT short-day; WT long-day-*CpCyc*^*−/−*^ long-day; WT short-day-*CpCyc*^*−/−*^ short-day, and *CpCyc*^*−/−*^ long-day- *CpCyc*^*−/−*^ short-day. For amplitudes, a one-way ANOVA was performed; in case of a significant difference, pairwise comparisons were performed using Welch’s two sample *t*-test, corrected for FDR ≤ 20%. For peak times, Watson’s two-sample test of homogeneity was used instead. For features significant in multiple groups, the differences between rhythmicity were assessed using the circacompare function. Circular boxplots were created using the package CircularBoxplots 0.1.2^57^. Correlation network analysis was performed using the igraph 2.2.2 and corrr 0.4.5 packages. Pearson correlations were calculated for all pairs of identified metabolites and corrected for FDR at a level of 10^−7^. This manuscript and the employed code were created without the use of generative AI.

## Supplementary information

Supplementary Information

## Data Availability

Data is available for download at https://doi.org/10.5281/zenodo.21643966.

## Code availability

Code is available for download at https://github.com/kleinomicslab.
