# Supplementary material for: The clock gene cycle synchronizes circadian metabolic oscillations uniquely in reproductively active Culex pipiens females
Source: NPJ Metab Health Dis. 2026 Oct 6;4:43. doi: 10.1038/s44324-026-00136-y (PMC13642261; doi:10.1038/s44324-026-00136-y)
Supplement: Supplementary file 1 [file 44324_2026_136_MOESM1_ESM.pdf]

Supplementary Materials

The clock gene *cycle* synchronizes circadian metabolic oscillations uniquely in reproductively active *Culex pipiens* females

Mizuki Yoshida[1,2], Matthew E. Wolkoff[2,3], Cheolho Sim[4], Megan E. Meuti[2], Matthias S. Klein[5,2*]

[1] Extremotolerance Biotechnology Group, Department of Biomaterial Sciences, Institute of Agrobiological Sciences, National Agriculture and Food Research Organization, 1-2 Owashi, Tsukuba, Ibaraki, Japan

[2] Department of Entomology, The Ohio State University, 2021 Coffey Road, Columbus, OH 43210, USA

[3] Department of Veterinary Biosciences, The Ohio State University, 1900 Coffey Road, Columbus, OH 43210, USA

[4] Department of Biology, Baylor University, 101 Bagby Ave, Waco, TX 76706, USA

[5] Department of Animal Science, McGill University, 21,111 Lakeshore Road, Ste.-Anne-de-Bellevue, QC, J7W0L7, Canada

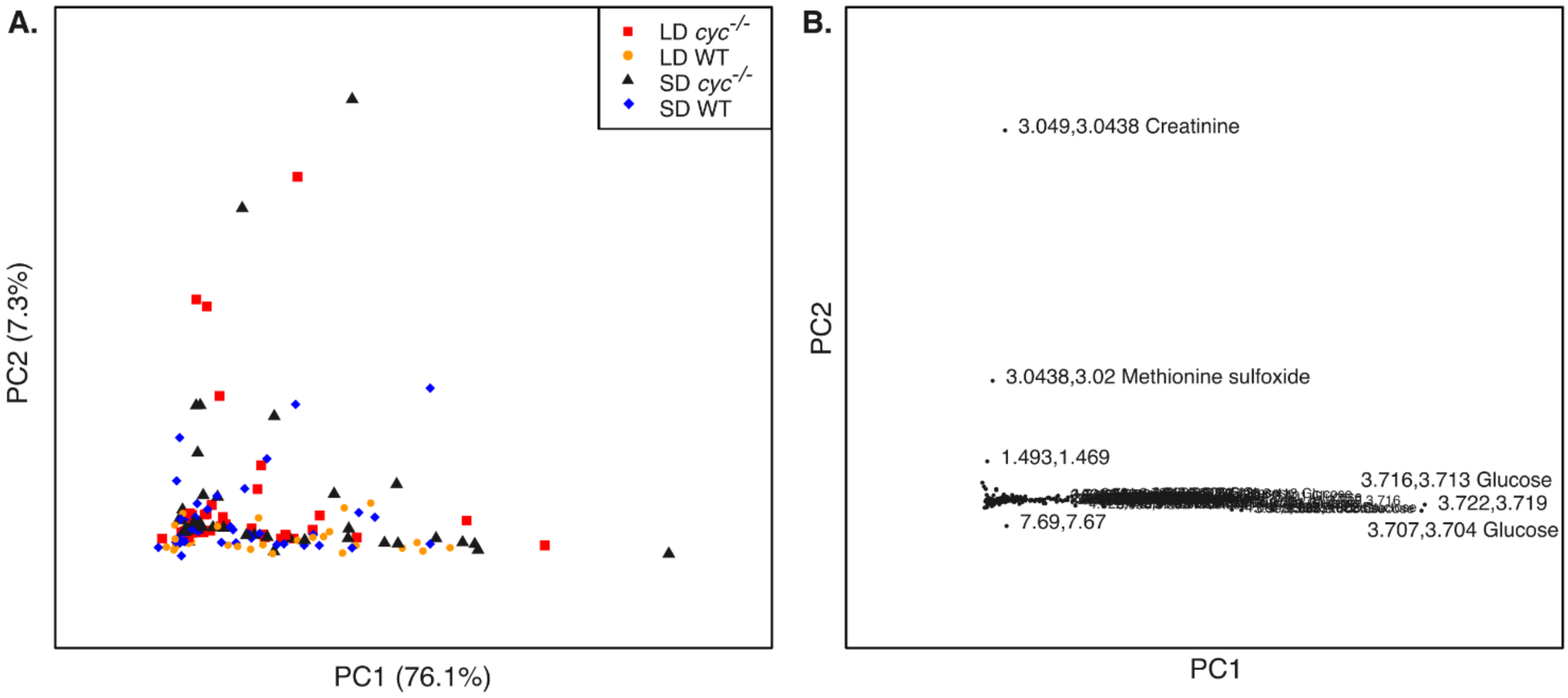

Supplementary Figure 1. A: Principal Component Analysis (PCA) scores plot of NMR spectra of aqueous extracts of mosquitoes showing partial separation of *CpCyc*$^{-/-}$ mutants. WT indicates wild-type mosquitoes, *cyc*$^{-/-}$ indicates *CpCyc*$^{-/-}$mutants, and LD and SD designate long and short photoperiods, respectively. B: PCA Loadings plot.

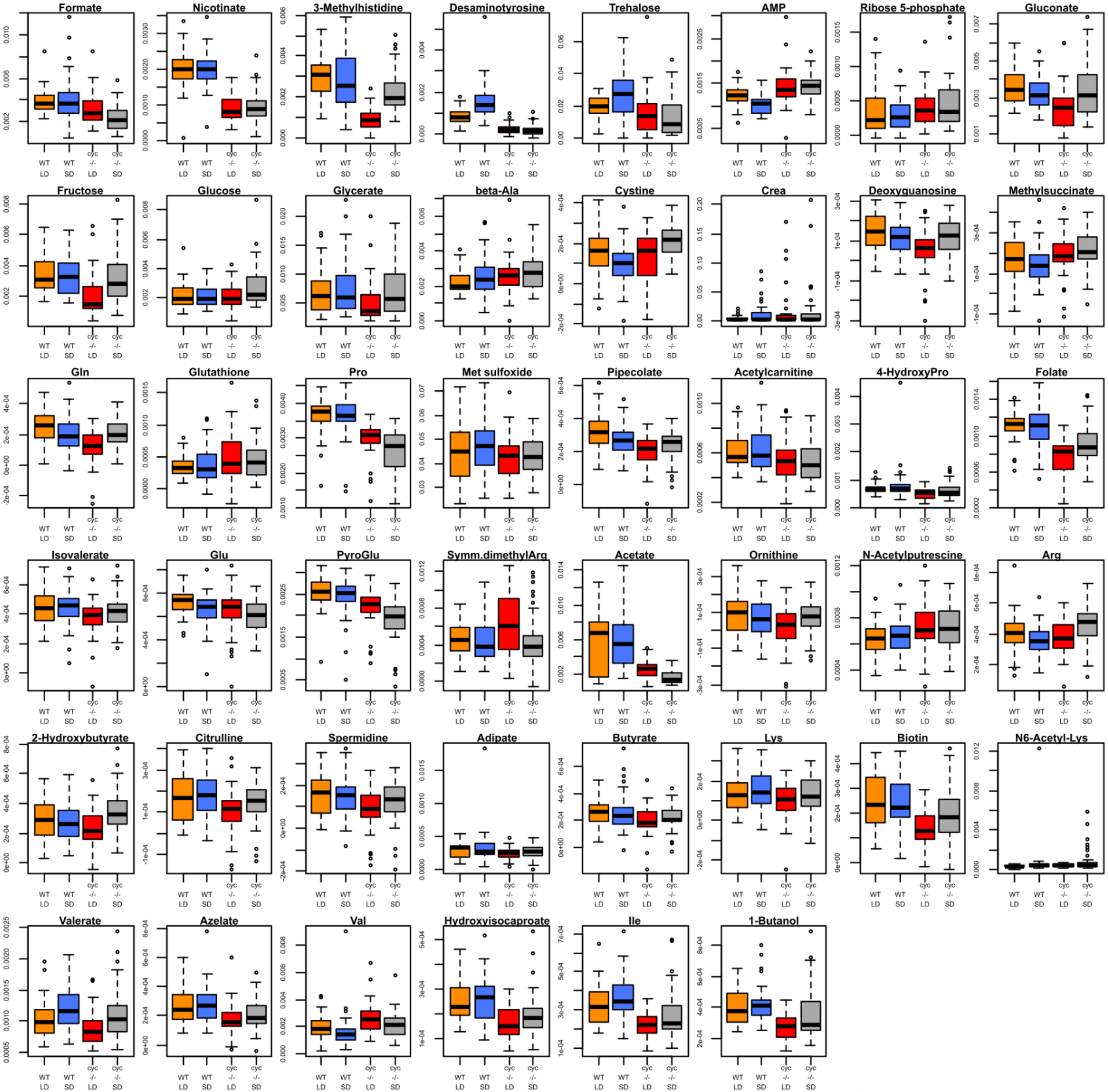

Supplementary Figure 2. Boxplots for metabolites that differ significantly between genotypes and/or photoperiods.

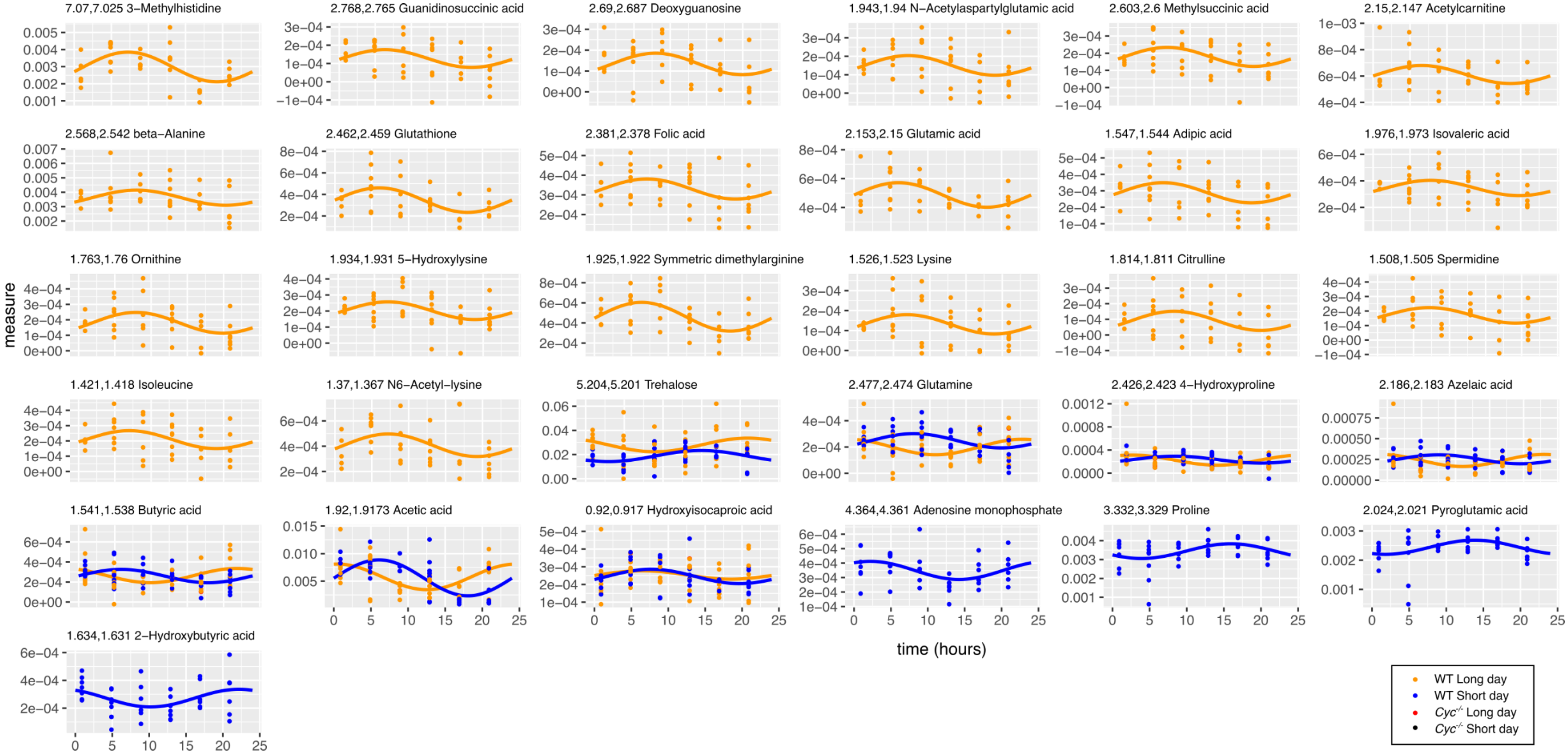

Supplementary Figure 3. Rhythmicity of significant metabolites for WT female mosquitoes reared in long day (orange) and short day (blue) conditions. Only significantly oscillating metabolites are shown.

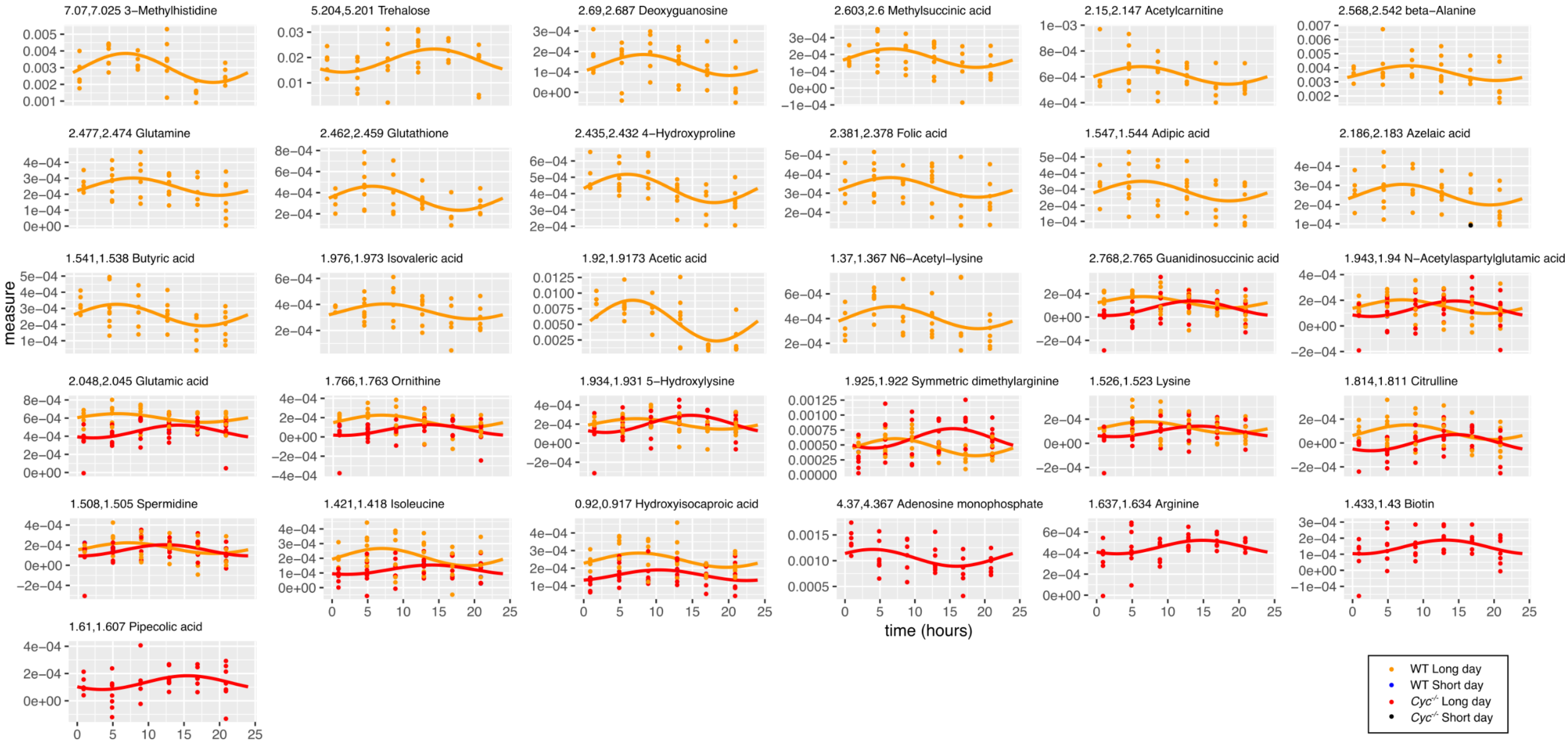

Supplementary Figure 4. Rhythmicity of significant metabolites for WT females (orange) and *CpCyc*$^{-/-}$ females (red) reared in long-day conditions. Only significantly oscillating metabolites are shown.

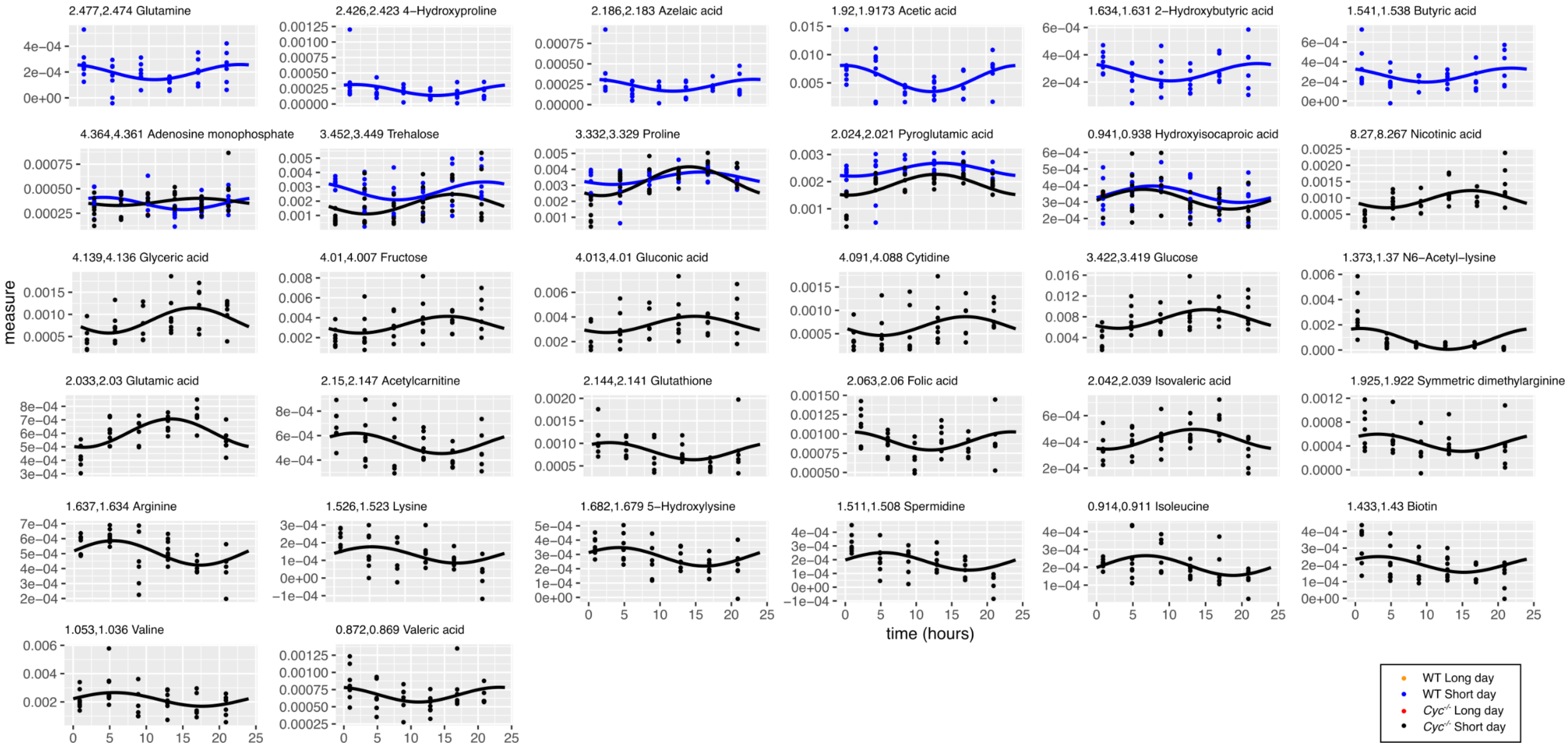

Supplementary Figure 5. Rhythmicity of significant metabolites for WT females (blue) vs. *CpCyc*$^{-/-}$ females (black) reared in short-day conditions. Only significantly oscillating metabolites are shown.

Supplementary Table 1. Correlation coefficients for metabolites differing significantly between genotypes and/or photoperiods. Asterisks (*) indicate significant correlations. Positive values indicate elevated in *CpCyc*$^{-/-}$ or in short-day (diapause-inducing) conditions.

Metabolite	Genotype	Photoperiod	Spectral area
1-Butanol	-0.72*	0.39*	0.887,0.884
2-Hydroxybutyric acid	0.06	0.41*	1.634,1.631
3-Methylhistidine	-0.97*	0.47*	7.07,7.025
4-Hydroxyproline	-0.47*	0.3*	2.126,2.123
Acetic acid	-1.23*	-0.15	1.92,1.9173
Acetylcarnitine	-0.48*	0.04	2.15,2.147
Adenosine monophosphate	0.82*	-0.25*	4.523,4.52
Adipic acid	-0.38*	0.16	1.547,1.544
Arginine	0.35*	0.22	1.64,1.637
Azelaic acid	-0.69*	0.14	1.244,1.241
beta-Alanine	0.34*	0.29*	3.184,3.17
Biotin	-0.76*	0.29*	1.424,1.421
Butyric acid	-0.45*	0.1	1.541,1.538
Citrulline	-0.5*	0.23	1.592,1.589
Creatinine	0.3*	0.17	3.049,3.0438
Cystine	0.38*	0.15	3.17,3.167
Deoxyguanosine	-0.47*	0.18	2.693,2.69
Desaminotyrosine	-1.28*	0.42*	6.915,6.885
Folic acid	-1.11*	0.28*	2.063,2.06
Formic acid	-0.71*	-0.19	8.462,8.456
Fructose	-0.48*	0.34*	4.01,4.007
Gluconic acid	-0.43*	0.26	4.013,4.01
Glucose	0.28*	0.36*	3.929,3.926
Glutamic acid	-0.41*	-0.3*	2.033,2.03
Glutamine	-0.55*	0.16	2.477,2.474
Glutathione	0.41*	-0.05	2.462,2.459
Glyceric acid	-0.24	0.27*	3.752,3.749
Hydroxyisocaproic acid	-0.77*	0.25*	0.92,0.917
Isoleucine	-0.86*	0.38*	0.896,0.893
Isovaleric acid	-0.34*	0.16	2.042,2.039
Lysine	-0.28*	0.27*	1.526,1.523
Methionine sulfoxide	-0.37*	0.14	2.325,2.21
Methylsuccinic acid	0.44*	-0.06	2.603,2.6
N6-Acetyl-lysine	0.11	0.4*	1.373,1.37

N-Acetylputrescine	0.47*	0.07	1.667,1.664
Nicotinic acid	-1.48*	0.02	8.27,8.267
Ornithine	-0.32*	0.15	1.805,1.802
Pipecolic acid	-0.72*	-0.02	2.18,2.177
Proline	-1.19*	-0.26*	2.354,2.351
Pyroglutamic acid	-0.88*	-0.39*	2.024,2.021
Ribose 5-phosphate	0.28*	-0.02	4.316,4.313
Spermidine	-0.46*	0.16	1.583,1.58
Symmetric dimethylarginine	0.27*	-0.32*	1.925,1.922
Trehalose	-0.63*	0.29*	5.204,5.201
Valeric acid	-0.37*	0.59*	1.301,1.298
Valine	0.54*	-0.32*	1.053,1.036

Supplementary Table 2. Features showing significant rhythmicity in WT females reared under long-day conditions. Peak time indicates the Zeitgeber time point at which the daily maximum concentration is observed.

Bin borders /Metabolite ID	p-value	Mesor	Amplitude	Peak time [hours]
**7.885,7.86**	0.0046	0.00064	0.00015	9.1
**7.07,7.025 3-Methylhistidine**	5.8e-05	0.003	0.00087	7.2
**5.213,5.21**	0.022	0.00017	8.5e-05	19
**5.204,5.201 Trehalose**	0.0059	0.019	0.0046	15
**5.201,5.198 Trehalose**	0.0088	0.014	0.0033	15
**5.198,5.195 Trehalose**	0.0034	0.0099	0.0027	15
**5.195,5.192 Trehalose**	0.028	0.00085	3e-04	16
**4.31,4.307**	0.024	0.00032	1e-04	23
**4.307,4.304**	0.015	0.00033	1e-04	21
**4.304,4.301**	0.013	0.00029	8.1e-05	22
**4.301,4.298**	0.0076	0.00039	0.00014	22
**4.298,4.295**	0.012	0.00031	0.00011	22
**4.295,4.292**	0.0035	0.00022	9.2e-05	22
**4.25,4.247**	0.018	0.00043	9.1e-05	23
**4.244,4.241**	0.009	0.00045	1e-04	23
**4.241,4.238**	0.0024	0.00042	0.00013	22
**3.749,3.746**	0.0047	0.0087	0.0025	2.6
**3.743,3.74**	0.012	0.0071	0.0019	2.3
**3.455,3.452 Trehalose**	0.0077	0.017	0.004	15
**3.443,3.44 Trehalose**	0.016	0.0089	0.0015	15
**3.311,3.308**	0.021	0.00022	5.3e-05	8.1
**3.278,3.275**	0.015	0.00097	0.00012	15
**3.218,3.215**	0.0023	0.00086	0.00021	6
**3.215,3.212**	0.001	0.00099	0.00023	7.3
**3.212,3.209 3-Methylhistidine**	0.015	9e-04	0.00013	5.5
**3.146,3.143**	0.0057	0.00012	6.3e-05	7.7
**3.14,3.137**	0.0025	2e-04	6.6e-05	7.1
**3.137,3.134**	0.0038	0.00035	0.00012	7.7
**3.134,3.131**	0.003	0.00058	0.00017	6.1
**3.128,3.125**	0.012	0.00072	0.00013	7.2
**3.125,3.122**	0.0035	0.00051	0.00015	6.4
**3.122,3.119**	0.011	0.00054	0.00016	7.6
**3.119,3.116**	0.00019	0.00044	0.00018	7.7
**3.116,3.113**	0.00014	0.00042	0.00014	6.1

**3.113,3.11**	0.021	0.00043	0.00011	6.7
**3.11,3.107**	3.9e-05	0.00033	0.00014	7.7
**3.107,3.104**	0.00013	0.00034	0.00013	6.3
**3.104,3.101**	0.011	0.00037	0.00011	6.1
**3.101,3.098**	0.012	0.00028	0.00011	7.4
**3.092,3.089**	0.01	0.00012	7.3e-05	7.5
**2.942,2.939**	0.025	0.00015	5.1e-05	7.4
**2.768,2.765 Guanidinosuccinic acid**	0.026	0.00013	4.9e-05	6.3
**2.69,2.687 Deoxyguanosine**	0.022	0.00013	5.2e-05	8
**2.681,2.678**	0.022	0.00011	5.3e-05	6.6
**2.66,2.657 N-Acetylaspartylglutamic acid**	0.026	7e-05	4.6e-05	6.9
**2.603,2.6 Methylsuccinic acid**	0.011	0.00018	5.5e-05	6.8
**2.6,2.597 Acetylcarnitine**	0.0013	0.00014	7.3e-05	6.6
**2.568,2.542 beta-Alanine**	0.027	0.0036	0.00052	8.4
**2.537,2.534**	0.0047	0.00022	5.7e-05	7.1
**2.504,2.501 Methylsuccinic acid**	0.027	0.00016	5.6e-05	6.4
**2.48,2.477 Acetylcarnitine**	0.0052	2e-04	5.6e-05	6.2
**2.477,2.474 Glutamine**	0.017	0.00025	5.4e-05	7.8
**2.474,2.471**	0.0087	0.00011	5.7e-05	7.5
**2.471,2.468 Glutathione**	1.6e-05	0.00029	0.00011	6.3
**2.465,2.462**	0.00069	0.00013	8.1e-05	7
**2.462,2.459 Glutathione**	0.00087	0.00035	0.00011	6
**2.453,2.45**	1.3e-05	0.00031	0.00015	6.2
**2.45,2.447**	0.032	0.00078	9.5e-05	5.4
**2.444,2.441**	0.0036	0.00023	7e-05	6.5
**2.441,2.438**	0.00013	0.00069	0.00019	5.6
**2.435,2.432 4-Hydroxyproline**	0.00021	0.00043	8.7e-05	6
**2.432,2.429**	0.0041	0.00055	9.9e-05	6.3
**2.426,2.423 4-Hydroxyproline**	0.016	0.00023	6e-05	7.7
**2.381,2.378 Folic acid**	0.024	0.00033	5.1e-05	7.1
**2.372,2.369 Folic acid**	0.03	0.0011	9.5e-05	5.6
**2.339,2.336 Glutamic acid**	0.0019	0.00034	6.3e-05	7.5
**2.33,2.327**	0.014	0.00024	5.4e-05	6.5
**2.327,2.325**	0.0018	0.00019	5.2e-05	7.3
**2.192,2.189 Adipic acid**	0.029	0.00017	5.1e-05	7.3
**2.186,2.183 Azelaic acid**	0.014	0.00025	5.4e-05	7.5
**2.183,2.18 Butyric acid**	0.004	0.00037	7.9e-05	6.5
**2.177,2.174**	0.0096	0.00038	7e-05	6.9
**2.174,2.171**	0.0076	0.00063	9.1e-05	6.1

**2.168,2.165 Glutamine**	9e-04	0.00035	8e-05	6.8
**2.165,2.162**	0.0087	0.00054	8.4e-05	5.8
**2.156,2.153**	0.011	0.00032	5.8e-05	6.3
**2.153,2.15 Glutamic acid**	0.00074	0.00049	8.5e-05	6
**2.15,2.147 Acetylcarnitine**	0.02	0.00061	6.8e-05	6.6
**2.12,2.117 4-Hydroxyproline**	0.018	0.00039	7.3e-05	7.1
**2.105,2.102**	0.029	0.00019	5.2e-05	7.8
**2.102,2.099**	0.011	0.00056	5.8e-05	5.1
**2.087,2.084**	0.023	0.00087	7.1e-05	4.2
**2.057,2.054 Glutamine**	0.013	0.00066	6e-05	5.5
**2.048,2.045 Glutamic acid**	0.029	6e-04	4.7e-05	5.7
**1.976,1.973 Isovaleric acid**	0.025	0.00035	5.8e-05	7.8
**1.952,1.949 Ornithine**	0.032	0.00012	5.7e-05	7.5
**1.943,1.94 N-Acetylaspartylglutamic acid**	0.024	0.00015	5.3e-05	7
**1.934,1.931 5-Hydroxylysine**	0.014	2e-04	5.5e-05	6.8
**1.928,1.925**	0.0051	0.00029	6.2e-05	7.2
**1.925,1.922 Symmetric dimethylarginine**	0.00015	0.00046	0.00014	6.4
**1.922,1.92**	0.00036	0.00059	0.00021	6.1
**1.92,1.9173 Acetic acid**	2.6e-06	0.0056	0.0033	6.1
**1.9173,1.916**	2.6e-05	0.00065	0.00025	6.4
**1.916,1.915**	0.0041	0.00035	8e-05	6.2
**1.841,1.838 Lysine**	0.032	0.00011	5.2e-05	6.2
**1.835,1.832**	0.033	0.00012	5.3e-05	7.6
**1.814,1.811 Citrulline**	0.031	9e-05	6.1e-05	7.8
**1.766,1.763 Ornithine**	0.012	0.00016	6.3e-05	6.9
**1.763,1.76 Ornithine**	0.0074	0.00018	6.7e-05	7.9
**1.754,1.751**	0.024	3e-04	6.1e-05	6.4
**1.748,1.745 Symmetric dimethylarginine**	0.0028	3e-04	8e-05	6.9
**1.703,1.7**	0.021	0.00036	7.2e-05	7.5
**1.565,1.562 Adipic acid**	0.021	0.00023	5.1e-05	6.3
**1.562,1.559 Adipic acid**	0.022	0.00028	5.5e-05	5.9
**1.55,1.547 Adipic acid**	0.0096	0.00032	6.8e-05	7
**1.547,1.544 Adipic acid**	0.026	0.00029	6.1e-05	6.7
**1.541,1.538 Butyric acid**	0.006	0.00026	6.7e-05	5.9
**1.538,1.535**	0.0081	0.00021	6e-05	6.5
**1.532,1.529 Lysine**	0.0068	0.00015	6.6e-05	5.9
**1.526,1.523 Lysine**	0.03	0.00013	4.8e-05	6.9
**1.523,1.52 Lysine**	0.0068	0.00014	5.9e-05	7.8
**1.52,1.517**	0.015	0.00015	5.1e-05	7.8

**1.508,1.505 Spermidine**	0.029	0.00017	5.3e-05	7.2
**1.469,1.466**	0.0046	0.00028	7.6e-05	6.5
**1.454,1.451**	0.031	0.00022	5.3e-05	6.6
**1.43,1.427 Isoleucine**	0.03	2e-04	5.2e-05	7.6
**1.421,1.418 Isoleucine**	0.023	0.00021	5.9e-05	7
**1.379,1.376**	0.022	3e-04	5.6e-05	6.4
**1.37,1.367 N6-Acetyl-lysine**	0.017	0.00041	8.9e-05	7.2
**1.367,1.364**	0.029	0.00028	6.1e-05	6.3
**1.163,1.16**	0.011	0.00017	5.3e-05	6.5
**1.154,1.151**	0.0026	0.00026	8.5e-05	6.9
**1.151,1.148**	3.6e-06	0.00095	0.00048	5.9
**1.148,1.145**	0.0036	3e-04	8.9e-05	6.6
**1.145,1.142**	9.7e-06	0.00045	0.00022	5.8
**1.142,1.139**	1.4e-05	0.00075	0.00033	6.1
**1.112,1.109**	0.00075	0.00036	0.00021	5.2
**0.92,0.917 Hydroxyisocaproic acid**	0.032	0.00025	4.1e-05	7.7
**0.848,0.845**	0.02	0.00014	4.2e-05	6.8

Supplementary Table 3. Features showing significant rhythmicity in *CpCyc*$^{-/-}$ mutants reared under long day conditions. Peak time indicates the Zeitgeber time point at which the daily maximum concentration is observed.

Bin borders /Metabolite ID	p-value	Mesor	Amplitude	Peak time [hours]
**8.355,8.32**	0.0044	0.0011	0.00028	12
**6.115,6.09**	0.013	0.0011	0.00021	11
**4.448,4.445**	0.011	0.00022	0.00012	10
**4.442,4.439**	0.025	0.00063	2e-04	9.6
**4.436,4.433**	0.0041	0.00029	0.00012	10
**4.37,4.367 Adenosine monophosphate**	0.015	0.0011	0.00016	3.9
**4.331,4.328**	0.029	0.00041	8.8e-05	4
**4.328,4.325**	0.026	0.00039	7.2e-05	4.1
**4.325,4.322**	0.03	0.00039	7.5e-05	4.7
**4.286,4.283**	0.01	0.00059	0.00012	9.1
**4.283,4.28**	0.02	0.00049	8.9e-05	8.8
**4.277,4.274**	0.012	0.00031	7.4e-05	11
**3.296,3.293**	0.018	9e-04	2e-04	16
**3.275,3.272**	0.022	0.0016	0.00028	2.4
**3.233,3.23**	0.0025	0.006	0.0012	2.2
**3.209,3.206**	0.018	0.00074	0.00023	1.2
**3.206,3.203**	0.0011	0.0014	0.00062	1.1
**3.128,3.125**	0.011	0.00065	0.00018	12
**3.119,3.116**	0.022	0.00016	7.9e-05	11
**2.768,2.765 Guanidinosuccinic acid**	0.017	7.5e-05	6.2e-05	13
**2.669,2.666**	0.021	0.00017	7.3e-05	14
**2.537,2.534**	0.028	9.5e-05	5.8e-05	15
**2.474,2.471**	0.011	0.00014	0.00011	15
**2.468,2.465**	0.011	0.00014	7.9e-05	14
**2.42,2.4**	0.028	0.016	0.0021	18
**2.399,2.396**	0.028	0.00013	5.8e-05	15
**2.396,2.393**	0.0082	0.00012	7.1e-05	15
**2.393,2.39 N-Acetylaspartylglutamic acid**	0.027	8.7e-05	5.8e-05	14
**2.384,2.381**	0.0032	8.7e-05	7.6e-05	16
**2.21,2.207**	0.021	9.6e-05	5.3e-05	15
**2.156,2.153**	0.03	0.00029	7.1e-05	16
**2.048,2.045 Glutamic acid**	0.017	0.00045	7.1e-05	14
**1.979,1.976 Ornithine**	0.016	2e-04	6.2e-05	15
**1.973,1.97 Lysine**	0.023	1e-04	6.6e-05	15

**1.949,1.946 Lysine**	0.017	9e-05	5.8e-05	16
**1.943,1.94 N-Acetylaspartylglutamic acid**	0.022	0.00013	6.1e-05	14
**1.94,1.937 N-Acetylaspartylglutamic acid**	0.0042	0.00016	7.3e-05	15
**1.937,1.934 N-Acetylaspartylglutamic acid**	0.019	0.00015	8e-05	14
**1.934,1.931 5-Hydroxylysine**	0.0037	2e-04	9e-05	14
**1.931,1.928 5-Hydroxylysine**	0.0024	0.00022	0.00012	15
**1.928,1.925**	0.015	0.00035	0.00013	14
**1.925,1.922 Symmetric dimethylarginine**	0.016	0.00061	0.00016	15
**1.9173,1.916**	0.032	0.00055	0.00012	13
**1.847,1.844 Lysine**	0.026	7.6e-05	4.6e-05	15
**1.844,1.841 Lysine**	0.026	6e-05	5.5e-05	15
**1.835,1.832**	0.0098	7.8e-05	7e-05	13
**1.814,1.811 Citrulline**	0.0051	2.6e-06	6.8e-05	15
**1.766,1.763 Ornithine**	0.031	7.1e-05	5.6e-05	13
**1.76,1.757**	0.0096	0.00014	6.5e-05	14
**1.757,1.754**	0.0064	0.00017	6.9e-05	14
**1.754,1.751**	0.019	0.00026	5.9e-05	16
**1.751,1.748**	0.0066	0.00019	6.3e-05	13
**1.742,1.739**	0.02	0.00033	7.9e-05	14
**1.733,1.73**	0.025	0.00063	1e-04	14
**1.691,1.688 Lysine**	0.012	0.00017	5.9e-05	13
**1.688,1.685**	0.011	2e-04	6.1e-05	14
**1.685,1.682 Lysine**	0.03	0.00017	3.9e-05	14
**1.682,1.679 5-Hydroxylysine**	0.0046	0.00029	8.3e-05	15
**1.679,1.676**	0.012	0.00033	5.9e-05	14
**1.673,1.67**	0.031	0.00076	0.00012	15
**1.637,1.634 Arginine**	0.032	0.00046	6.4e-05	15
**1.628,1.625 Biotin**	0.032	0.00021	4.6e-05	16
**1.619,1.616 Spermidine**	0.031	0.00013	4.4e-05	16
**1.613,1.61 Citrulline**	0.021	0.00012	4.8e-05	12
**1.61,1.607 Pipecolic acid**	0.032	0.00013	5e-05	15
**1.592,1.589 Citrulline**	0.032	0.00011	5e-05	15
**1.577,1.574 Spermidine**	0.028	0.00014	4.4e-05	15
**1.52,1.517**	0.019	0.00012	5.4e-05	15
**1.517,1.514 Lysine**	0.016	0.00011	5.8e-05	14
**1.508,1.505 Spermidine**	0.026	0.00015	5.5e-05	12
**1.499,1.496**	0.0041	0.00022	7e-05	11
**1.448,1.445 Isoleucine**	0.025	0.00015	4.1e-05	15
**1.445,1.442 Isoleucine**	0.022	0.00018	4.6e-05	13

**1.442,1.439 Isoleucine**	0.011	0.00013	5.3e-05	14
**1.436,1.433 Isoleucine**	0.015	0.00014	4.5e-05	15
**1.433,1.43 Biotin**	0.031	0.00015	4.3e-05	13
**1.418,1.415 Isoleucine**	0.03	0.00013	3.6e-05	15
**1.412,1.409 Isoleucine**	0.0029	0.00017	4.3e-05	15
**1.391,1.388**	0.011	0.00014	4.8e-05	17
**1.388,1.385**	0.025	0.00016	4.2e-05	16
**1.379,1.376**	0.00048	0.00018	6.6e-05	17
**1.265,1.262 Isoleucine**	0.0053	0.00033	7.3e-05	15
**1.262,1.259**	0.014	0.00036	7.2e-05	13
**1.247,1.244**	0.023	0.00019	4.6e-05	14
**1.226,1.223**	0.028	0.00024	5.4e-05	13
**0.941,0.938 Hydroxyisocaproic acid**	0.031	3e-04	4.8e-05	8.7
**0.932,0.929 Hydroxyisocaproic acid**	0.018	2e-04	4.3e-05	7.5
**0.917,0.914 Hydroxyisocaproic acid**	0.029	0.00017	3.2e-05	12
**0.884,0.881**	0.032	0.00031	4.3e-05	14
**0.851,0.848**	0.0041	0.00012	5e-05	14
**0.848,0.845**	0.011	1e-04	4.7e-05	14

Supplementary Table 4. Features showing significant rhythmicity in WT females reared under short-day conditions. Peak time indicates the Zeitgeber time point at which the daily maximum concentration is observed.

Bin borders /Metabolite ID	p-value	Mesor	Amplitude	Peak time [hours]
**6.115,6.09**	0.015	0.00043	0.00017	6.9
**5.45,5.447**	0.013	1e-04	5.8e-05	3.6
**4.448,4.445**	0.0033	9.8e-05	9.2e-05	7
**4.439,4.436**	5.1e-05	0.00022	0.00012	4.9
**4.436,4.433**	0.00021	0.00017	9.1e-05	4.4
**4.394,4.391**	0.0066	0.00016	7e-05	3.9
**4.391,4.388**	0.015	0.00027	7.2e-05	3.3
**4.388,4.385**	0.011	0.00028	6.8e-05	4.4
**4.385,4.382**	0.015	0.00022	5.5e-05	3.8
**4.382,4.379**	4e-04	0.00018	8.5e-05	3.4
**4.364,4.361 Adenosine monophosphate**	0.01	0.00035	6.2e-05	2.2
**4.361,4.358**	0.0091	1e-04	6e-05	2.9
**4.358,4.355**	0.00048	0.00024	8.3e-05	2.8
**4.355,4.352**	0.00035	1e-04	7.5e-05	4
**4.352,4.349**	0.0027	0.00017	5.7e-05	4.1
**4.34,4.337**	0.0037	8.7e-05	7.6e-05	3.1
**4.337,4.334**	0.00057	5.9e-05	8e-05	3.8
**4.334,4.331**	0.024	0.00013	6.6e-05	2.6
**4.331,4.328**	0.0082	0.00013	6.7e-05	3
**4.328,4.325**	0.0077	0.00011	7.5e-05	4.4
**4.325,4.322**	0.0072	0.00016	7.4e-05	3.9
**4.31,4.307**	0.0092	0.00027	0.00014	4.4
**4.301,4.298**	0.0059	0.00038	0.00019	3
**4.298,4.295**	0.00092	0.00027	0.00015	3
**4.295,4.292**	0.0011	0.00016	9.1e-05	3.8
**4.286,4.283**	0.015	0.00033	9.1e-05	3.9
**4.283,4.28**	0.0018	0.00025	9.8e-05	4.1
**4.277,4.274**	0.0049	0.00019	7.2e-05	3.9
**4.265,4.262**	0.03	0.00029	5.9e-05	3.7
**4.25,4.247**	0.018	0.00038	8.8e-05	5.3
**4.247,4.244**	0.016	0.00041	8.4e-05	4.5
**4.244,4.241**	0.0028	0.00036	0.00011	5.3
**4.241,4.238**	0.0059	0.00035	0.00012	4.4
**4.151,4.148**	0.032	0.00036	0.00019	1.8

**3.944,3.941**	0.026	0.0024	0.00039	2.2
**3.452,3.449 Trehalose**	0.025	0.0027	0.00062	21
**3.35,3.347**	0.0053	0.00071	0.00017	12
**3.347,3.344 Proline**	0.018	0.0046	0.00054	14
**3.341,3.338 Proline**	0.016	0.0023	0.00024	14
**3.338,3.335 Proline**	0.015	0.0028	3e-04	15
**3.335,3.332 Proline**	0.03	0.0011	0.00013	13
**3.332,3.329 Proline**	0.013	0.0034	0.00039	16
**3.308,3.305**	0.018	0.00021	5.3e-05	5.5
**3.305,3.302**	0.028	0.00022	4.9e-05	6.8
**3.233,3.23**	0.00038	0.005	0.00088	20
**3.23,3.227**	0.0077	0.0037	0.00052	18
**3.2,3.197**	0.008	0.0021	0.00052	4.7
**3.146,3.143**	0.027	7.8e-05	5e-05	2.7
**3.092,3.089**	0.0067	0.00011	9.4e-05	0.98
**2.645,2.642**	0.028	0.00012	5.9e-05	1.2
**2.477,2.474 Glutamine**	0.022	2e-04	5.9e-05	23
**2.426,2.423 4-Hydroxyproline**	0.032	0.00022	8.8e-05	1.7
**2.351,2.348 Proline**	0.027	0.0027	0.00027	16
**2.186,2.183 Azelaic acid**	0.031	0.00024	7.3e-05	23
**2.147,2.144**	0.003	0.001	0.00017	4.8
**2.09,2.087 Proline**	0.032	0.0014	0.00014	15
**2.084,2.081 Proline**	0.02	0.0041	0.00043	15
**2.075,2.072 Proline**	0.021	0.003	0.00029	16
**2.069,2.066 Proline**	0.027	0.0031	0.00029	16
**2.06,2.057 Proline**	0.025	0.0018	0.00017	17
**2.03,2.027 Pyroglutamic acid**	0.02	0.0041	0.00043	15
**2.024,2.021 Pyroglutamic acid**	0.023	0.0024	0.00024	14
**2.021,2.018 Pyroglutamic acid**	0.013	0.0049	0.00052	16
**2.018,2.015 Pyroglutamic acid**	0.03	0.0032	0.00031	15
**2.015,2.012 Pyroglutamic acid**	0.027	0.0028	0.00028	15
**2.012,2.009 Pyroglutamic acid**	0.021	0.0024	0.00025	16
**2.009,2.006 Pyroglutamic acid**	0.018	0.0047	5e-04	15
**2,1.997 Proline**	0.019	0.0033	0.00035	15
**1.991,1.988 Proline**	0.016	0.0013	0.00013	16
**1.92,1.9173 Acetic acid**	0.00034	0.0058	0.0023	0.52
**1.9173,1.916**	0.0074	0.00067	0.00017	0.42
**1.727,1.724**	0.018	0.00093	0.00011	6.2
**1.721,1.718**	0.02	8e-04	8.6e-05	5.5

**1.634,1.631 2-Hydroxybutyric acid**	0.022	0.00027	6.4e-05	22
**1.625,1.622**	0.023	0.00018	5e-05	21
**1.541,1.538 Butyric acid**	0.029	0.00026	7.1e-05	22
**1.493,1.469**	0.0017	0.022	0.0052	5.9
**1.151,1.148**	0.00015	0.0011	0.00048	24
**1.148,1.145**	0.013	0.00031	6.6e-05	22
**1.145,1.142**	0.0012	0.00049	0.00018	24
**1.142,1.139**	0.00072	0.00087	3e-04	23
**1.121,1.118**	0.021	0.00019	5.1e-05	15
**0.941,0.938 Hydroxyisocaproic acid**	0.026	0.00035	5e-05	7.7
**0.746,0.743**	0.0095	0.00022	3.9e-05	16

Supplementary Table 5. Features showing significant rhythmicity in *CpCyc*$^{-/-}$ mutants reared under short day conditions. Peak time indicates the Zeitgeber time point at which the daily maximum concentration is observed.

Bin borders /Metabolite ID	p-value	Mesor	Amplitude	Peak time [hours]
**7.885,7.86**	0.0046	0.00064	0.00015	9.1
**7.07,7.025 3-Methylhistidine**	5.8e-05	0.003	0.00087	7.2
**5.213,5.21**	0.022	0.00017	8.5e-05	19
**5.204,5.201 Trehalose**	0.0059	0.019	0.0046	15
**5.201,5.198 Trehalose**	0.0088	0.014	0.0033	15
**5.198,5.195 Trehalose**	0.0034	0.0099	0.0027	15
**5.195,5.192 Trehalose**	0.028	0.00085	3e-04	16
**4.31,4.307**	0.024	0.00032	1e-04	23
**4.307,4.304**	0.015	0.00033	1e-04	21
**4.304,4.301**	0.013	0.00029	8.1e-05	22
**4.301,4.298**	0.0076	0.00039	0.00014	22
**4.298,4.295**	0.012	0.00031	0.00011	22
**4.295,4.292**	0.0035	0.00022	9.2e-05	22
**4.25,4.247**	0.018	0.00043	9.1e-05	23
**4.244,4.241**	0.009	0.00045	1e-04	23
**4.241,4.238**	0.0024	0.00042	0.00013	22
**3.749,3.746**	0.0047	0.0087	0.0025	2.6
**3.743,3.74**	0.012	0.0071	0.0019	2.3
**3.455,3.452 Trehalose**	0.0077	0.017	0.004	15
**3.443,3.44 Trehalose**	0.016	0.0089	0.0015	15
**3.311,3.308**	0.021	0.00022	5.3e-05	8.1
**3.278,3.275**	0.015	0.00097	0.00012	15
**3.218,3.215**	0.0023	0.00086	0.00021	6
**3.215,3.212**	0.001	0.00099	0.00023	7.3
**3.212,3.209 3-Methylhistidine**	0.015	9e-04	0.00013	5.5
**3.146,3.143**	0.0057	0.00012	6.3e-05	7.7
**3.14,3.137**	0.0025	2e-04	6.6e-05	7.1
**3.137,3.134**	0.0038	0.00035	0.00012	7.7
**3.134,3.131**	0.003	0.00058	0.00017	6.1
**3.128,3.125**	0.012	0.00072	0.00013	7.2
**3.125,3.122**	0.0035	0.00051	0.00015	6.4
**3.122,3.119**	0.011	0.00054	0.00016	7.6
**3.119,3.116**	0.00019	0.00044	0.00018	7.7
**3.116,3.113**	0.00014	0.00042	0.00014	6.1

**3.113,3.11**	0.021	0.00043	0.00011	6.7
**3.11,3.107**	3.9e-05	0.00033	0.00014	7.7
**3.107,3.104**	0.00013	0.00034	0.00013	6.3
**3.104,3.101**	0.011	0.00037	0.00011	6.1
**3.101,3.098**	0.012	0.00028	0.00011	7.4
**3.092,3.089**	0.01	0.00012	7.3e-05	7.5
**2.942,2.939**	0.025	0.00015	5.1e-05	7.4
**2.768,2.765 Guanidinosuccinic acid**	0.026	0.00013	4.9e-05	6.3
**2.69,2.687 Deoxyguanosine**	0.022	0.00013	5.2e-05	8
**2.681,2.678**	0.022	0.00011	5.3e-05	6.6
**2.66,2.657 N-Acetylaspartylglutamic acid**	0.026	7e-05	4.6e-05	6.9
**2.603,2.6 Methylsuccinic acid**	0.011	0.00018	5.5e-05	6.8
**2.6,2.597 Acetylcarnitine**	0.0013	0.00014	7.3e-05	6.6
**2.568,2.542 beta-Alanine**	0.027	0.0036	0.00052	8.4
**2.537,2.534**	0.0047	0.00022	5.7e-05	7.1
**2.504,2.501 Methylsuccinic acid**	0.027	0.00016	5.6e-05	6.4
**2.48,2.477 Acetylcarnitine**	0.0052	2e-04	5.6e-05	6.2
**2.477,2.474 Glutamine**	0.017	0.00025	5.4e-05	7.8
**2.474,2.471**	0.0087	0.00011	5.7e-05	7.5
**2.471,2.468 Glutathione**	1.6e-05	0.00029	0.00011	6.3
**2.465,2.462**	0.00069	0.00013	8.1e-05	7
**2.462,2.459 Glutathione**	0.00087	0.00035	0.00011	6
**2.453,2.45**	1.3e-05	0.00031	0.00015	6.2
**2.45,2.447**	0.032	0.00078	9.5e-05	5.4
**2.444,2.441**	0.0036	0.00023	7e-05	6.5
**2.441,2.438**	0.00013	0.00069	0.00019	5.6
**2.435,2.432 4-Hydroxyproline**	0.00021	0.00043	8.7e-05	6
**2.432,2.429**	0.0041	0.00055	9.9e-05	6.3
**2.426,2.423 4-Hydroxyproline**	0.016	0.00023	6e-05	7.7
**2.381,2.378 Folic acid**	0.024	0.00033	5.1e-05	7.1
**2.372,2.369 Folic acid**	0.03	0.0011	9.5e-05	5.6
**2.339,2.336 Glutamic acid**	0.0019	0.00034	6.3e-05	7.5
**2.33,2.327**	0.014	0.00024	5.4e-05	6.5
**2.327,2.325**	0.0018	0.00019	5.2e-05	7.3
**2.192,2.189 Adipic acid**	0.029	0.00017	5.1e-05	7.3
**2.186,2.183 Azelaic acid**	0.014	0.00025	5.4e-05	7.5
**2.183,2.18 Butyric acid**	0.004	0.00037	7.9e-05	6.5
**2.177,2.174**	0.0096	0.00038	7e-05	6.9
**2.174,2.171**	0.0076	0.00063	9.1e-05	6.1

**2.168,2.165 Glutamine**	9e-04	0.00035	8e-05	6.8
**2.165,2.162**	0.0087	0.00054	8.4e-05	5.8
**2.156,2.153**	0.011	0.00032	5.8e-05	6.3
**2.153,2.15 Glutamic acid**	0.00074	0.00049	8.5e-05	6
**2.15,2.147 Acetylcarnitine**	0.02	0.00061	6.8e-05	6.6
**2.12,2.117 4-Hydroxyproline**	0.018	0.00039	7.3e-05	7.1
**2.105,2.102**	0.029	0.00019	5.2e-05	7.8
**2.102,2.099**	0.011	0.00056	5.8e-05	5.1
**2.087,2.084**	0.023	0.00087	7.1e-05	4.2
**2.057,2.054 Glutamine**	0.013	0.00066	6e-05	5.5
**2.048,2.045 Glutamic acid**	0.029	6e-04	4.7e-05	5.7
**1.976,1.973 Isovaleric acid**	0.025	0.00035	5.8e-05	7.8
**1.952,1.949 Ornithine**	0.032	0.00012	5.7e-05	7.5
**1.943,1.94 N-Acetylaspartylglutamic acid**	0.024	0.00015	5.3e-05	7
**1.934,1.931 5-Hydroxylysine**	0.014	2e-04	5.5e-05	6.8
**1.928,1.925**	0.0051	0.00029	6.2e-05	7.2
**1.925,1.922 Symmetric dimethylarginine**	0.00015	0.00046	0.00014	6.4
**1.922,1.92**	0.00036	0.00059	0.00021	6.1
**1.92,1.9173 Acetic acid**	2.6e-06	0.0056	0.0033	6.1
**1.9173,1.916**	2.6e-05	0.00065	0.00025	6.4
**1.916,1.915**	0.0041	0.00035	8e-05	6.2
**1.841,1.838 Lysine**	0.032	0.00011	5.2e-05	6.2
**1.835,1.832**	0.033	0.00012	5.3e-05	7.6
**1.814,1.811 Citrulline**	0.031	9e-05	6.1e-05	7.8
**1.766,1.763 Ornithine**	0.012	0.00016	6.3e-05	6.9
**1.763,1.76 Ornithine**	0.0074	0.00018	6.7e-05	7.9
**1.754,1.751**	0.024	3e-04	6.1e-05	6.4
**1.748,1.745 Symmetric dimethylarginine**	0.0028	3e-04	8e-05	6.9
**1.703,1.7**	0.021	0.00036	7.2e-05	7.5
**1.565,1.562 Adipic acid**	0.021	0.00023	5.1e-05	6.3
**1.562,1.559 Adipic acid**	0.022	0.00028	5.5e-05	5.9
**1.55,1.547 Adipic acid**	0.0096	0.00032	6.8e-05	7
**1.547,1.544 Adipic acid**	0.026	0.00029	6.1e-05	6.7
**1.541,1.538 Butyric acid**	0.006	0.00026	6.7e-05	5.9
**1.538,1.535**	0.0081	0.00021	6e-05	6.5
**1.532,1.529 Lysine**	0.0068	0.00015	6.6e-05	5.9
**1.526,1.523 Lysine**	0.03	0.00013	4.8e-05	6.9
**1.523,1.52 Lysine**	0.0068	0.00014	5.9e-05	7.8
**1.52,1.517**	0.015	0.00015	5.1e-05	7.8

**1.508,1.505 Spermidine**	0.029	0.00017	5.3e-05	7.2
**1.469,1.466**	0.0046	0.00028	7.6e-05	6.5
**1.454,1.451**	0.031	0.00022	5.3e-05	6.6
**1.43,1.427 Isoleucine**	0.03	2e-04	5.2e-05	7.6
**1.421,1.418 Isoleucine**	0.023	0.00021	5.9e-05	7
**1.379,1.376**	0.022	3e-04	5.6e-05	6.4
**1.37,1.367 N6-Acetyl-lysine**	0.017	0.00041	8.9e-05	7.2
**1.367,1.364**	0.029	0.00028	6.1e-05	6.3
**1.163,1.16**	0.011	0.00017	5.3e-05	6.5
**1.154,1.151**	0.0026	0.00026	8.5e-05	6.9
**1.151,1.148**	3.6e-06	0.00095	0.00048	5.9
**1.148,1.145**	0.0036	3e-04	8.9e-05	6.6
**1.145,1.142**	9.7e-06	0.00045	0.00022	5.8
**1.142,1.139**	1.4e-05	0.00075	0.00033	6.1
**1.112,1.109**	0.00075	0.00036	0.00021	5.2
**0.92,0.917 Hydroxyisocaproic acid**	0.032	0.00025	4.1e-05	7.7
**0.848,0.845**	0.02	0.00014	4.2e-05	6.8

Supplementary Table 6. Metabolites exhibiting significant rhythmicity in this study and their roles in mosquito metabolic pathways.

Metabolite	Oscillating in WT short-day	Oscillating in WT long-day	Oscillating in CpCyc-/- short-day	Oscillating in CpCyc-/- long-day	Pathways/Role
**2-Hydroxybutyric acid**	Yes				Propanoate metabolism
**3-Methylhistidine**		Yes			Histidine metabolism
**4-Hydroxyproline**	Yes	Yes			Arginine and proline metabolism, D-Amino acid metabolism, ABC transporters
**5-Hydroxylysine**		Yes	Yes	Yes	Lysine degradation
**Acetic acid**	Yes	Yes			Glycolysis / Gluconeogenesis, Taurine and hypotaurine metabolism, Phosphonate and phosphinate metabolism, Glycosaminoglycan biosynthesis - heparan sulfate / heparin, Pyruvate metabolism, Glyoxylate and dicarboxylate metabolism, Sulfur metabolism
**Acetylcarnitine**		Yes	Yes		Insulin resistance
**Adenosine monophosphate**	Yes		Yes	Yes	Purine metabolism, FoxO signaling pathway, mTOR signaling pathway
**Adipic acid**		Yes			Caprolactam degradation, Microbial metabolism in diverse environments, Degradation of aromatic compounds
**Arginine**			Yes	Yes	Arginine biosynthesis, Arginine and proline metabolism, D-Amino acid metabolism, Aminoacyl-tRNA biosynthesis, ABC transporters, mTOR signaling pathway
**Azelaic acid**	Yes	Yes			Pyrimidine metabolism
**beta-Alanine**		Yes			Pyrimidine metabolism, beta-Alanine metabolism, Propanoate metabolism, Pantothenate and CoA biosynthesis, Neuroactive ligand-receptor interaction
**Biotin**			Yes	Yes	Biotin metabolism, ABC transporters
**Butyric acid**	Yes	Yes			Butanoate metabolism
**Citrulline**		Yes		Yes	Arginine biosynthesis
**Cytidine**			Yes		Pyrimidine metabolism
**Deoxyguanosine**		Yes			Purine metabolism, ABC transporters
**Folic acid**		Yes	Yes		One carbon pool by folate, Folate biosynthesis
**Fructose**			Yes		Fructose and mannose metabolism, Galactose metabolism, Starch and sucrose metabolism, ABC transporters
**Gluconic acid**			Yes		Pentose phosphate pathway
**Glucose**			Yes		Glycolysis / Gluconeogenesis, Pentose phosphate pathway, Galactose metabolism, Starch and sucrose

					metabolism, Amino sugar and nucleotide sugar metabolism, ABC transporters, FoxO signaling pathway
**Glutamic acid**		Yes	Yes	Yes	Arginine biosynthesis, Alanine, aspartate and glutamate metabolism, Arginine and proline metabolism, Histidine metabolism, Taurine and hypotaurine metabolism, D-Amino acid metabolism, Glutathione metabolism, Glyoxylate and dicarboxylate metabolism, Butanoate metabolism, Porphyrin metabolism, Nitrogen metabolism, Aminoacyl-tRNA biosynthesis, ABC transporters, FoxO signaling pathway, Neuroactive ligand-receptor interaction
**Glutamine**	Yes	Yes			Arginine biosynthesis, Purine metabolism, Pyrimidine metabolism, Alanine, aspartate and glutamate metabolism, D-Amino acid metabolism, Glyoxylate and dicarboxylate metabolism, Vitamin B6 metabolism, Nitrogen metabolism, Aminoacyl-tRNA biosynthesis, ABC transporters
**Glutathione**		Yes	Yes		Cysteine and methionine metabolism, Glutathione metabolism, ABC transporters
**Glyceric acid**			Yes		Pentose phosphate pathway, Glycine, serine and threonine metabolism, Glycerolipid metabolism, Glyoxylate and dicarboxylate metabolism
**Guanidinosuccinic acid**		Yes		Yes	L-arginine metabolism
**Hydroxyisocaproic acid**	Yes	Yes	Yes	Yes	L-leucine metabolism
**Isoleucine**		Yes	Yes	Yes	Valine, leucine and isoleucine degradation, Valine, leucine and isoleucine biosynthesis, Aminoacyl-tRNA biosynthesis, ABC transporters
**Isovaleric acid**		Yes	Yes		Biosynthesis of alkaloids derived from histidine and purine, Biosynthesis of secondary metabolites, Protein digestion and absorption
**Lysine**		Yes	Yes	Yes	Lysine degradation, D-Amino acid metabolism, Biotin metabolism, Aminoacyl-tRNA biosynthesis, ABC transporters
**Methylsuccinic acid**		Yes			L-isoleucine metabolism
**N-Acetylaspartyl-glutamic acid**		Yes		Yes	Neurotransmitter
**N6-Acetyl-lysine**		Yes	Yes		Lysine degradation
**Nicotinic acid**			Yes		Nicotinate and nicotinamide metabolism

**Ornithine**		Yes		Yes	Arginine biosynthesis, Arginine and proline metabolism, D-Amino acid metabolism, Glutathione metabolism, ABC transporters
**Pipecolic acid**				Yes	Lysine degradation
**Proline**	Yes		Yes		Arginine and proline metabolism, D-Amino acid metabolism, Aminoacyl-tRNA biosynthesis, ABC transporters
**Pyroglutamic acid**	Yes		Yes		Glutathione metabolism
**Spermidine**		Yes	Yes	Yes	Arginine and proline metabolism, beta-Alanine metabolism, Glutathione metabolism, ABC transporters
**Symmetric dimethylarginine**		Yes	Yes	Yes	Protein catabolism
**Trehalose**	Yes	Yes	Yes		Starch and sucrose metabolism, ABC transporters
**Valeric acid**			Yes		Microbial metabolism, Gut-brain axis
**Valine**			Yes		Valine, leucine and isoleucine degradation, Valine, leucine and isoleucine biosynthesis, Pantothenate and CoA biosynthesis, Aminoacyl-tRNA biosynthesis, ABC transporters
